# Patterns and Associated Factors of Physical Activity among Adolescents in Nigeria

**DOI:** 10.1371/journal.pone.0150142

**Published:** 2016-02-22

**Authors:** Adewale L. Oyeyemi, Cornelius M. Ishaku, Jameela Oyekola, Hajara D. Wakawa, Aliyu Lawan, Safira Yakubu, Adetoyeje Y. Oyeyemi

**Affiliations:** 1 Department of Physiotherapy, College of Medical Sciences, University of Maiduguri, Maiduguri, Nigeria; 2 Physical Activity, Sport and Recreation Research Focus Area, Faculty of Health Sciences, North-West University, Potchefstroom, South Africa; 3 Industrial Clinic, Nigerian National Petroleum Commission Kaduna Medical Area, Kaduna, Nigeria; 4 Department of Physiotherapy, Federal Medical Center, Birnin Kebbi, Kebbi State, Nigeria; University of Hawaii Cancer Center, UNITED STATES

## Abstract

**Introduction:**

Understanding the context where adolescents’ physical activity (PA) takes place could impact a more targeted approach to implement PA promotion and interventions in Africa. However, standardized data on adolescents’ PA behaviour is lacking in Nigeria. We described PA patterns in the various domains (home, school, transport, leisure-time) and intensity categories (light-intensity PA, moderate- to vigorous- intensity physical activity [MVPA] and total PA), and their associations with sociodemographic factors and socioeconomic status (SES) among secondary school adolescents in Nigeria.

**Methods:**

A cross-sectional survey was conducted in a representative sample of 1006 secondary school adolescents (12–18 years, 50.4% girls) in Maiduguri, Nigeria. Self-reported PA was assessed with an adapted version of the Activity Questionnaire for Adolescents and Young Adults. Outcomes were weekly minutes (min/wk) of PA spent in the various domains and intensity categories. Multivariate ANOVA was used to examine associations of PA scores (domains and intensity levels) with adolescents’ sociodemographic characteristics and SES, and track differences in PA scores between subgroups.

**Results:**

The total sample reported most PA at school (1525 min/wk), the least during active transportation (210 min/wk), and only 37% engaged in 60 min of MVPA daily. Boys reported significantly more leisure-time PA (*P*<0.001), active transportation (*P*<0.001), MVPA (*P* = 0.023) and total PA (*P* = 0.003) than girls, while girls reported more school-based PA (*P* = 0.009), home-based PA (*P*<0.001) and light-intensity PA (*P*<0.001) than boys. Moderate-intensity PA (*P* = 0.024) and total PA (*P* = 0.049) were significantly higher in younger age group than in older group. Household car ownership was associated with less active transportation (*P* = 0.009), less moderate-intensity PA (*P* = 0.048) and with more leisure-time PA (*P* = 0.013). High parental SES was associated with more leisure-time PA (*P* = 0.002), more MVPA (*P* = 0.047) and less active transportation (*P*<0.001). Adolescents of various weight status differed significantly in their leisure-time PA (*P*<0.001), moderate-intensity PA (*P* = 0.011) and total PA (*P* = 0.033).

**Conclusions:**

The patterns and levels of physical activity among adolescents in Nigeria vary according to the adolescents’ age, gender, weight status and SES. These findings have important public health implications for identifying subgroups of Nigerian adolescents that should be targeted for effective physical activity promoting interventions.

## Introduction

Sufficient physical activity is associated with substantial health benefits in young people that can track into adulthood [1–4]. Yet, an astounding majority of adolescents in both the developed and developing countries [5,6] do not meet the health related guidelines of engaging in at least 60 minutes of moderate-to vigorous- physical activity (MVPA) daily [3,4]. In the African region, only 8% to 35% of African youth engaged in sufficient levels of physical activity for 60 minutes a day on at least 5 days per week [6]. In Nigeria, about 72% of school going adolescents reported engaging in physical activity at least once a month [7], 59% engaged at moderate levels [8], and more than 50% engaged in low levels of physical activity [9]. However, no study was found that provided estimates on the proportion of Nigerian youth meeting the health-related recommended guidelines for sufficient levels of physical activity, and there is no data on the patterns and domains of physical activity among adolescents and children in Nigeria [10].

While current health-related physical activity guidelines expect youth to be physically active in all domains of living [3,4], majority of studies in Africa have focused mainly on adolescents’ overall physical activity levels with little or no report on physical activity context [6–20]. Because adolescents can be active in different domains (at home, at school, during transport and leisure time), identifying the contexts where physical activity take place could provide useful information for effective physical activity promotion in this age group [21–23]. Addressing this knowledge gap remains a top research priority for low- and middle income- countries (LMICs) where the understanding of evidence-based strategies for increasing physical activity is poor [24–27]. Moreover, such evidence could broaden the options for physical activity intervention programmes and contribute to public health efforts to control the rising mortality and morbidity of non-communicable diseases (NCDs) now occurring in many African countries [28–30].

Examining country-specific population-based data on youth’s physical activity patterns and the domains where they occur could be critical for effective planning and interventions that are appropriate and relevant to local context, especially in Nigeria where such data are scarce. With an estimated population of about 53.5 million youth (projected to be more than double at 116.2 million by the year 2050), Nigeria has the highest population of youth aged between 10–24 years in the entire African continent [31], and physical inactivity related NCDs mortalities are increasingly rising in this country [32]. Thus, availability of baseline national or subnational data on physical activity patterns and associated factors in adolescents could be considered an urgent public health issue for Nigeria. Such information would be useful for future surveillance and could help to identify Nigerian adolescents’ populations and subgroups at risk of inactivity related morbidity and mortality. Therefore, the objective of this study was to describe the patterns in specific domains (home, school, transport, leisure time) and intensity categories (light-physical activity, moderate-physical activity and vigorous-physical activity) of self-reported physical activity in a subnational sample of secondary school adolescents in Maiduguri, Nigeria. A secondary objective was to explore the associations between the adolescents’ physical activity patterns, and sociodemographic factors and socioeconomic status (SES).

## Methods

### Design and Participants

This was a descriptive cross-sectional study in a subnational sample of secondary school adolescents in Nigeria. Information on participants’ recruitment, sampling strategy, study procedure and measures has been described in detail elsewhere [33]. Briefly, multistage sampling technique was used to select participants from secondary schools stratified by area level income of their neighborhood location (high-income and low-income schools) and by school types (public and private schools) (stage 1). In the second stage of sampling, one or two classes of about 45 students were randomly selected (lottery method) each from the second to the sixth grade in 11 selected secondary schools (5 private and 6 public schools). Thereafter, 20 students were randomly recruited through ballot from each of the selected classes (70 classes in total). All students in the 2^nd^ to 6^th^ grade of study and aged between 12 and 18 years were eligible to participate in the survey. Study questionnaires were filled out anonymously by the adolescents while in the classroom. In total, 1006 adolescents (71.8% response rate) attending secondary schools in the metropolitan city of Maiduguri, Nigeria provided complete and useable data for the study. Maiduguri is the capital and largest city of Borno State in North Eastern Nigeria, with area of 72.6 km^2^ and a population of about 732,600 inhabitants [34,35].

### Ethics Statement

The study has been conducted in accordance to the principles expressed in the 1964 declaration of Helsinki and its later amendments. Written assent and written informed consent were respectively, obtained from all the participants and their parents through a cover letter distributed few days prior to questionnaire administration. On the test date, participants were informed that they could withdraw their assent or parental consent anytime if they were no longer interested in participating in the study or changed their mind. Permission was also obtained from the management of each of the schools before data collection, and the consent procedures and study protocol were approved by the Research and Ethics Committee of the University of Maiduguri Teaching Hospital, Nigeria, (AD/TH/EC/75).

### Measures

#### Assessment of physical activity

An adapted version of the Activity Questionnaire for Adolescents and Young Adults (AQuAA) was used to assess participants’ self-reported physical activity. Adaptation made was addition of examples of various intensities of activity and physical activity behaviours that are common among adolescents in Nigeria to those already on the original questionnaire. The AQuAA assessed physical activity done in the last 7 days in the domains of school, household, leisure time (including sport), active transportation to and from school and sedentary behaviours in leisure time. It estimated the time spent on light, moderate and vigorous intensity activities in terms of frequency (days per week) and duration (minutes per day) in each of the physical activity domain, except for active transportation. Outcomes were computed weekly minutes (min/wk) of physical activity scores in the different domains (school-based physical activity, home-based physical activity, leisure-time physical activity and transport-related physical activity) and intensity categories (light-intensity activity, moderate-intensity activity, vigorous-intensity activity, MVPA and total physical activity). The MVPA was computed by summing the time (min/wk) in moderate-intensity and vigorous-intensity activities across the domains. The reliability (ICCs = 0.44–0.59) and validity (ρ = 0.05–0.23) of AQuAA were fair to moderate [36,37], and are comparable to those of other adolescents’ self-report physical activity questionnaires [38–40]. The adapted version of the questionnaire has acceptable evidence of test-retest reliability (ICCs = 0.38–0.71) among adolescents in Nigeria [33].

#### Sociodemographic Characteristics

A short sociodemographic form was used to obtain information on age, gender, household ownership of cars, grade of study and parental educational and occupation level. Household ownership of cars was categorized into none or one or more cars in household. Information on parents’ education and occupation was used to compute participants’ family SES levels [41]. Parental educational status was classified (0–3) as no education, primary education, secondary education and tertiary education, for both father and mother. Responses to questions on parental occupational status were coded (0–3) into unemployed, retired/not working, blue collar job and white collar job, for both father and mother. Family SES was computed by summing the scores for parental education and occupation to produce a composite scale (0–16) which was further categorized into three groups as low (0–7), middle (8–10) and high (11–16) Family SES [41].

#### Anthropometric measurements

The height and weight of all the participants were measured before administering the questionnaire, following standardized procedure. The weight recorded to nearest 0.1 kg and height to nearest 0.1 cm were measured in light clothing using a digital scale and stadiometer. The body mass index (BMI) of the adolescents was calculated as body weight divided by the square of height (kg/m^2^). International age- and gender specific cut-points were used to assess BMI category (underweight, normal weight, overweight and obese) [42,43].

### Statistical analysis

All analyses were conducted in SPSS 18.0 for Windows. To limit unrealistic high values and based on previous studies of adolescents using a comparable questionnaire [22,44], physical activity scores were truncated in the different domains (school: max 1800 min/week; home: max 1680 min/week; transport time: max 1290 min/week; leisure time: max 1680 min/week; total physical activity: max 4200 min/week [10 hrs/day]) as well as in the different intensity levels (max 2100 min/week for light physical activity, and max of 1260 min/week for moderate physical activity and vigorous physical activity, respectively). School-based physical activity was truncated for fifty-six participants, home-based physical activity for thirty-eight, light-intensity physical activity for twenty-one, moderate-intensity physical activity for three and total physical activity for thirty-four participants. No truncation was done for any participant on transportation-related physical activity, leisure-time physical activity and vigorous-intensity physical activity. Because the physical activity variables (min/wk of domains and intensity levels) were non-normally distributed (skewed), log-transformation of the original variables was used in the analyses to improve normality. Raw data were used to calculate the descriptive statistics (mean, standard deviation, median and interquartile range) of the physical activity scores in the different domains and intensity levels for the total sample, gender groups, age categories, weight status categories, household car ownerships and family SES categories shown in the Result section and Tables.

A repeated-measure ANOVA was conducted each to compare the different domain scores (school, home transport, leisure time) and the different intensity scores (light-physical activity, moderate-physical activity, vigorous-physical activity, MVPA, total PA) in the total sample. Multivariate ANOVA was conducted to explore the associations between physical activity scores (domains and intensity levels) and each of the independent variables (gender, age groups, weight status, household car ownership and family SES categories). When between-subjects tests were significant, additional *Turkey post hoc* were conducted to track the differences in physical activity scores between subgroups. An α level of *P< 0*.*05* was used to determine the statistical significance.

## Results

The participants comprised of 1006 adolescents (50.4% girls) with a mean age of 15.3 ± 1.7 years and body mass index of 19.5 ± 3.4kg/m^2^. The majority of the adolescents reported household ownership of one or more cars (72.6%), were aged between 15 and 18 years (60.4%), were in the junior secondary school grade (55.3%), have ‘normal’ weight status (55.3%) and came from the middle or higher SES class (58.3%). Compared to boys, the girls were more likely to be overweight or obese (*p<*0.001) and being in the junior grades (*p*<0.001) (Table 1).

**Table 1:** Descriptive characteristics of the sample.

Variables	Total Sample (N = 1006)	Girls (n = 507, 50.4%)	Boys (n = 499, 49.6%)	*P*-values†
Age (years)	15.3 ± 1.7	15.1 ± 1.5	15.6 ± 1.9	<0.001*
Age group (n, %)				0.060
11–14	398 (39.6)	186 (36.7)	212 (42.5)	
15–18	608 (60.4)	321 (63.3)	287 (57.5)	
BMI (Kg/m^2^)	19.5 ± 3.4	19.8 ± 3.4	19.1 ± 3.3	0.007*
Weight status (n, %)				<0.001*
Underweight	412 (41.0)	144 (28.4)	268 (53.7)	
Normal weight	556 (55.3)	341 (67.3)	217 (43.1)	
Overweight	23 (2.3)	13 (2.6)	10 (2.0)	
Obese	15 (1.5)	9 (1.8)	6 (1.2)	
Ethnicity (n, %)				0.200
Igbo	91 (9.0)	54 (10.7)	37 (7.4)	
Hausa/Fulani	131 (13.0)	58 (11.4)	73 (14.6)	
Yoruba	54 (5.4)	30 (5.9)	24 (4.8)	
Kanuri	250 (25.0)	121 (23.9)	130 (26.1)	
Others	479 (47.6)	244 (48.1)	235 (47.1)	
Grade (n, %)				<0.001*
JSS Two	255 (25.3)	126 (24.9)	129 (25.9)	
JSS Three	302 (30.0)	201 (39.6)	101 (20.2)	
SSS One	133 (13.3)	80 (15.8)	54 (10.8)	
SSS Two	191 (19.0)	54 (10.7)	137 (27.5)	
SSS Three	124 (12.3)	46 (9.1)	78 (15.5)	
Household cars (n, %)				0.199
None	276 (27.4)	130 (25.6)	146 (29.3)	
One or more cars	730 (72.6)	377 (74.4)	353 (70.7)	
Family SES (n, %)				0.065
Low SES	419 (41.7)	193 (38.1)	226 (45.3)	
Middle SES	311 (31.1)	166 (32.7)	147 (29.5)	
High SES	274 (27.4)	148 (29.2)	126 (25.3)	

^†^- Values based on independent t-tests statistics for continuous variables and chi-Square Statistics for categorical variables;

*- Significant difference between samples (*P*<0.05); JSS- Junior Secondary School; SSS- Senior Secondary School; BMI- Body Mass Index; SES- Socioeconomic status.

About 37% (95% CI: 34%–40%) of the Nigerian adolescents fulfilled the recommended guidelines of 60 min per day of MVPA. Boys were more physically active than girls to meet the MVPA recommendations (54.3% vs 45.7%, *P* = 0.023), at leisure time (68 min/day vs 14 min/day, *P*<0.001), during active transportation (34 min/day vs 26 min/day, *P*<0.001) and in total physical activity (360 min/day vs 341 min/day, *P* = 0.003), while girls reported significantly more school-based physical activity (*P* = 0.009), home-based physical activity (*P*<0.001) and light intensity physical activity (*P*<0.001) than boys (Table 2).

**Table 2:** Participants’ physical activity per domain and intensity for total sample and by gender.

	Total Sample (N = 1006)	Girls (n = 507)	Boys (n = 499)	*P*-values†
**Domain of PA**				
School-based PA (min/wk)				0.009*
Mean	1525	1529	1522	
SD	303	296	310	
Median	1570	1575	1570	
Interquartile range	1515, 1650	1520, 1695	1521, 1655	
Home-based PA (min/wk)				<0.001*
Mean	649	734	565	
SD	522	520	512	
Median	500	595	390	
Interquartile range	240, 990	300, 1160	160, 877	
Transport-related PA (min/wk)				<0.001*
Mean	210	185	237	
SD	72	144	194	
Median	150	100	150	
Interquartile range	100, 300	100, 250	100, 300	
Leisure-time PA (min/wk)				<0.001*
Mean	288	100	476	
SD	276	103	310	
Median	120	30	360	
Interquartile range	100, 360	10, 120	120, 600	
**Intensity Level**				
Light PA (min/wk)				<0.001*
Mean	1403	1460	1345	
SD	361	366	348	
Median	1300	1340	1270	
Interquartile range	1235, 1582	1235, 1620	1205, 1480	
Moderate PA (min/wk)				<0.001*
Mean	280	243	316	
SD	286	278	289	
Median	192	140	220	
Interquartile range	75, 450	80, 470	50, 420	
Vigorous PA (min/wk)				0.627
Mean	250	247	253	
SD	241	235	246	
Median	180	180	180	
Interquartile range	91, 420	100, 445	83, 390	
MVPA (min/wk)				0.010*
Mean	550	424	584	
SD	420	404	429	
Median	400	350	450	
Interquartile range	229, 864	279, 915	192, 775	
Sufficient MVPA (n, %)				0.023*
No	630 (62.6)	335 (52.3)	295 (46.8)	
Yes	376 (37.4)	172 (45.7)	204 (54.3)	
Total PA (min/wk)				0.003*
Mean	2454	2390	2519	
SD	686	660	707	
Median	2380	2325	2445	
Interquartile range	2010, 3040	1990, 2993	2052, 3073	

^†^—Values based on independent t-tests statistics for continuous variables and chi-Square Statistics for categorical variables;

*—Significant difference between samples (*p*<0.05); PA—Physical Activity;

MVPA—Moderate-to-vigorous physical activity; min/wk—Minutes per week; SD—Standard Deviation.

**Fig 1:**
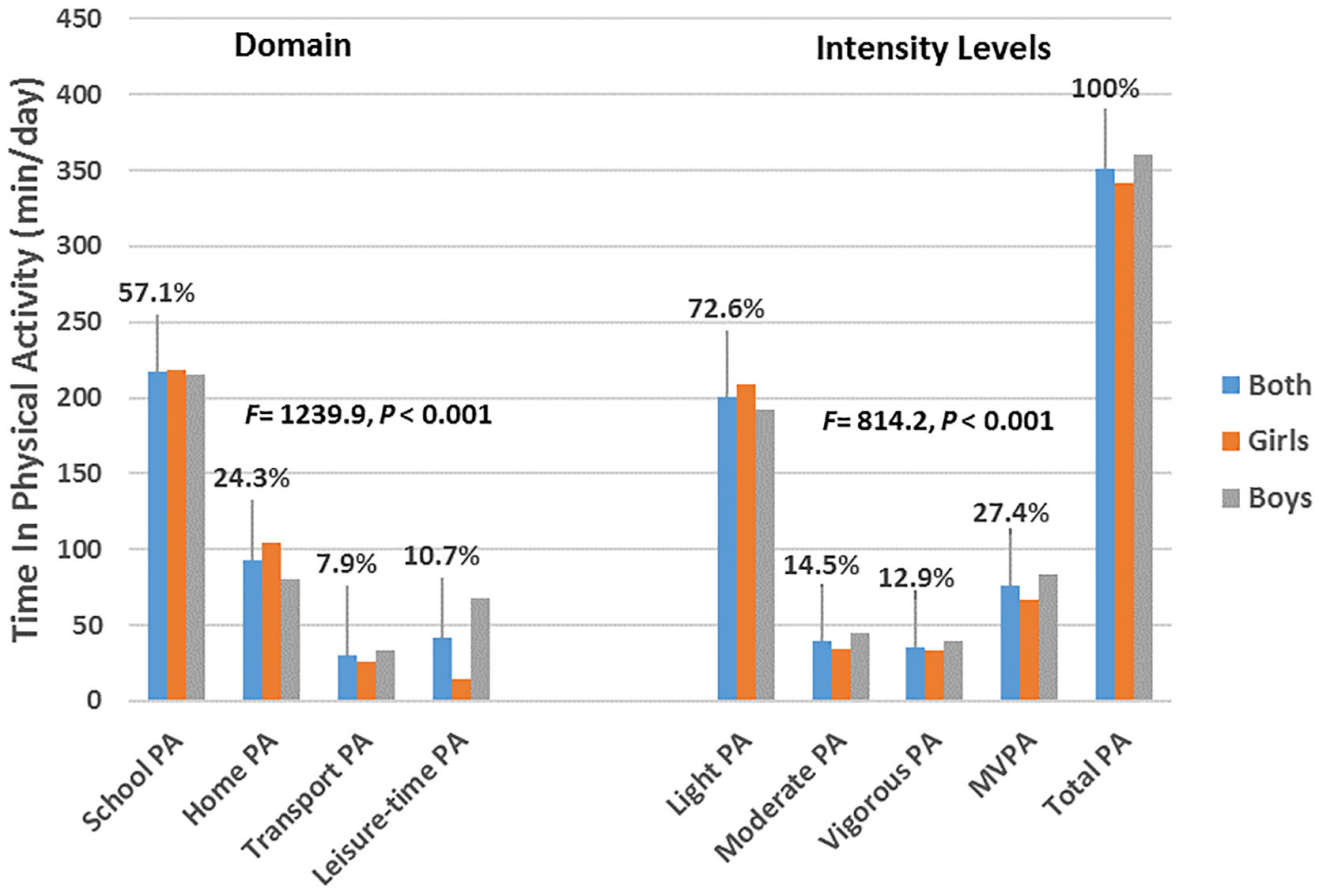
Proportion of time in domain and intensity levels of physical activity.

**Fig 2:**
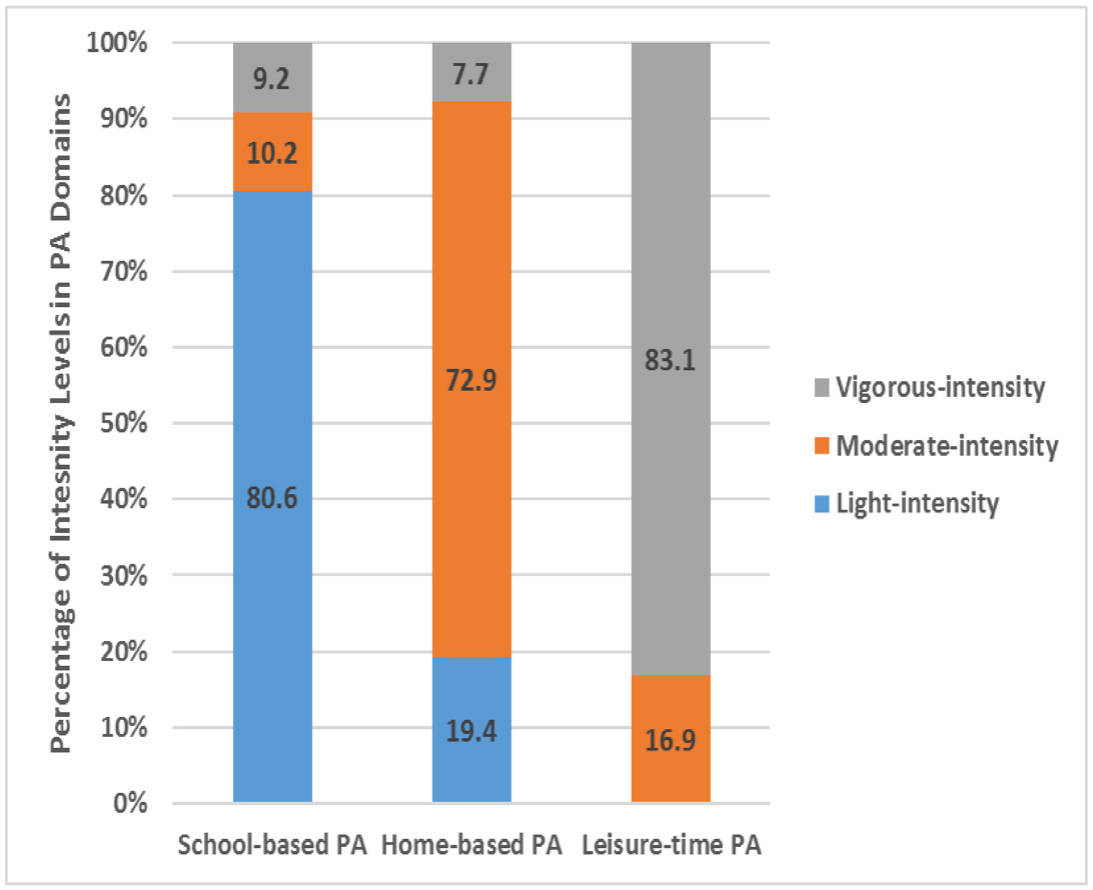
Proportion of intensity levels of activity reported in each domain of physical activity.

The total sample reported most physical activity at school (average about 217 min/day or 57% of sum of all domains), less at home (93 min/day or 24%), and the smallest amount of physical activity was reported during active transportation (30 min/day or 8%) and leisure-time (41 min/day or 11%). All adolescents reported on the average about 200 min/day (about 73% of all intensity levels) of light-intensity physical activity, 40 min/day (about 14% of all intensity levels) of moderate-intensity physical activity and 36 min/day (about 13% of all intensity levels) of vigorous-intensity physical activity (Fig 1 and Table 2). Most light intensity levels of activity were performed at school (81%), most moderate-intensity levels of activity at home (73%) and most vigorous-intensity levels of activity accumulated during leisure-time (83%) (Fig 2).

Age was significantly associated with moderate-intensity and total physical activity. The younger group (12–14 years) reported more moderate-intensity (*F* = 5.129, *P* = 0.024) and total physical activity (*F* = 2.893, *P* = 0.049) than the older group (15–18 years). Significant associations of body weight status were found with leisure-time physical activity (*F* = 8.855, *P*<0.001), moderate-intensity physical activity (*F* = 3.741, *P* = 0.011) and total physical activity (*F* = 2.919, *P* = 0.033). *Post hoc* analyses revealed that underweight adolescents reported more leisure time physical activity than normal weight (*P*<0.001) and obese (*P* = 0.011) participants. Overweight participants reported significantly lower moderate-intensity activity than the normal weight (*P* = 0.032) and underweight adolescents (*P* = 0.013). Furthermore, total physical activity was significantly lower in obese adolescents compared with their normal weight (*P* = 0.026) and underweight (*P* = 0.018) counterparts. Household car ownership was significantly associated with active transportation (*F* = 7.416, *P* = 0.007), leisure-time physical activity (*F* = 5.480, *P* = 0.019) and moderate-intensity physical activity (*F* = 4.324, *P* = 0.038). Adolescents reporting one or more household cars reported less duration of active transportation (*P* = 0.009) and moderate-intensity activity (*P* = 0.048) but more leisure-time activity (*P* = 0.013) compared to those without household car. Adolescents’ family SES was significantly associated with active transportation (*F* = 6.383, *P* = 0.002), leisure-time physical activity (*F* = 7.111, *P* = 0.001), moderate-intensity physical activity (*F* = 3.474, *P* = 0.031), MVPA (*F* = 2.578, *P* = 0.050) and total physical activity (*F* = 3.666, *P* = 0.026). Active transportation was higher among those with low (*P*<0.001) and medium (*P* = 0.005) familial SES than among adolescents with high familial SES. In contrast, leisure-time physical activity was greater among adolescents with high familial SES than those with medium (*P* = 0.002) and low (*P* = 0.003) familial SES. Also, adolescents with higher familial SES reported significantly more MVPA (*P* = 0.047) and total physical activity (*P* = 0.019) compared to those with low familial SES (Table 3).

**Table 3:** Physical activity per domain and intensity for groups based on age category, weight status, household cars and family SES.

		PA domain (min/week)				PA intensity (min/week)				
		School-based PA	Home-based PA	Transport-related PA	Leisure-time PA	Light PA	Moderate PA	Vigorous PA	MVPA	Total PA
Age (years)										
12–14	Mean	1520	651	213	234	1403	286	252	538	2457
	SD	318	522	183	270	383	283	238	421	690
	Median	1580	500	137	132	1290	200	180	400	4375
15–18	Mean	1528	647	207	230	1403	256	219	475	2442
	SD	293	523	164	280	346	288	243	428	684
	Median	1560	502	150	120	1302	190	177	410	2385
	*P-values*	0.785	0.480	0.318	0.343	0.942	**0.024**	0.088	0.157	**0.049**
Weight Status										
Underweight	Mean	1525	606	220	286	1372	266	242	508	2469
	SD	302	511	190	297	349	273	236	419	692
	Median	1570	437	150	180	1270	160	170	370	2387
Normal	Mean	1531	676	198	194	1424	293	255	549	2447
	SD	297	528	153	252	362	295	244	429	671
	Median	1575	527	130	100	1308	210	180	430	2370
Overweight	Mean	1526	759	260	213	1505	230	272	502	2539
	SD	264	548	230	282	409	292	264	472	728
	Median	1550	720	150	60	1560	70	200	350	2515
Obese	Mean	1313	639	325	153	1290	241	227	469	2144
	SD	513	536	206	281	504	271	228	392	954
	Median	1500	430	300	100	1290	210	135	330	1895
	*P-values*	0.601	0.952	0.287	**<0.001**	0.054	**0.011**	0.191	0.103	**0.033**
Household Car										
None	Mean	1496	662	217	231	1384	290	251	542	2446
	SD	336	541	171	291	369	284	236	421	725
	Median	1550	500	150	120	1305	212	180	439	2456
One or more	Mean	1536	644	206	232	1410	275	249	525	2456
	SD	289	515	173	270	358	287	242	427	671
	Median	1580	502	150	120	1294	180	175	380	2372
	*P-values*	0.225	0.313	**0.007**	**0.019**	0.843	**0.038**	0.867	0.170	0.929
Family SES										
Low	Mean	1497	624	228	220	1387	258	238	496	2401
	SD	339	531	180	273	390	282	237	420	701
	Median	1560	455	150	120	1270	150	165	360	2325
Medium	Mean	1525	659	204	225	1400	283	262	546	2456
	SD	301	519	189	278	353	282	250	424	688
	Median	1570	525	100	225	1305	210	190	420	2375
High	Mean	1567	674	189	256	1429	308	254	562	2531
	SD	237	512	147	276	322	294	237	433	654
	Median	1585	527	150	175	1340	210	180	439	2480
	*P-values*	0.125	0.123	**0.002**	**0.001**	0.114	**0.031**	0.311	**0.050**	**0.026**

PA—Physical Activity; MVPA—Moderate-to-vigorous physical activity; SES—Socioeconomic Status; min/week—Minutes per week; SD—Standard Deviation.

## Discussion

This study examined the contexts and intensity levels of self-reported physical activity in an urban population of secondary school adolescents in Nigeria, and explored the associations of the adolescents’ physical activity patterns with sociodemographic and SES factors. The main finding was that secondary school adolescents in Nigeria get most of their total minutes of physical activity at school (about 4hrs/day), followed by home (1.5hrs/day), and the least physical activity during active transportation to and from school (about 0.5hr/day) and during leisure-time (about 1hr/day). Leisure-time physical activity, active transportation, moderate-intensity activity and total physical activity were the main physical activity variables associated with Nigerian adolescents’ sociodemographic and SES factors (age, gender, weight status, household car ownership and family SES).

Only few studies that report on the domains and patterns of adolescents’ physical activity could be found to directly compare our findings [22,23,45]. The few available studies on contexts of adolescents’ physical activity have reported mixed findings. While US adolescents’ reported greater proportion of their physical activity (exercise and walking) at school and outdoors [23], European adolescents reported leisure-time as the most frequent context to engage in physical activity and the least context being home-based physical activity [22]. However, partly consistent with our findings, participation in physical education activities at school was a frequently reported physical activity context by majority of rural youth in South Africa [45,46]. Also, our result on school being the most common context for Nigerian adolescents’ physical activity seems to affirm the finding of a recent qualitative study that school is a common and an important outlet for South African adolescents to engage in physical activity [47]. It seems the promotion of extramural activities and provision of sports facilities and equipment at schools could be a viable approach to improve physical activity behaviours of school going adolescents in Africa.

In contrast to our findings of least physical activity in the contexts of leisure-time and active transportation, South African adolescents reported higher sport participation at leisure and spent more time in active transportation to and from school [45]. The difference in these findings may be due to the fact that the South African study was among rural adolescents who have little or no restriction to engage in active play at leisure-time and may have no choice rather than using active transport to and from school [45]. For urban population adolescents as in our study, the effects of urbanization may restrict the choice of active- leisure play and could favour more motorized mode of transportation to and from school [14,15]. Corroboratively, more than 70% of the adolescents in our study reported household ownership of one or more cars, and active transportation to and from school was significantly lower among this group compared to those without household ownership of car. All these taken together confirm the shifting patterns of physical activity in Africa from a more traditionally energy dependent to a more energy saving one, and support the emerging evidence of an ongoing physical activity transition in Africa [14–16].

The present results on the intensity of physical activity showed that light intensity activity contributed more to adolescents’ total physical activity than moderate-and vigorous- intensity activities, and more than 60% of Nigerian adolescents did not meet the international recommendations of engaging in 60 minutes of MVPA per day. Although youth in Africa may engage in large volumes of light and incidental moderate-intensity physical activity during domestic activities and active transportation [12,16,18], multiple studies have consistently reported majority of adolescents in Africa to be insufficiently active to meet the MVPA recommendations [6, 16–20]. Perhaps, the global physical activity recommendations that focus exclusively on MVPA may be misguided for adolescents in low resource settings of Africa [48]. Compared to youth in western high income countries [49,50], African youth could fail to meet the international physical activity guidelines because of accumulating lower MVPA but they could be more physically active (total volumes of physical activity) because of the very high volumes of light-intensity physical activity they accumulate daily [18]. Because light intensity activity is common among African youth [12,17,18,51], offers substantially more daily opportunities for physical activity engagement than the other activity intensities [52] and could be potentially related to health outcomes [53], there is need to reconsider the applicability of the current global guidelines to African youth. Contributing to the development of appropriate guidelines for African youth, future studies could focus on quantifying the health-related dosage or benefits of the relatively large volumes of light intensity physical activity common among African adolescents.

Our results showed that girls on the average spent approximately 23 min/day lower time in MVPA compared to boys (60.6 min/day vs 83.4 min/day), and moderate-intensity and total physical activity was lower in the older adolescents than in the younger group. Although some contrasting African findings exist on gender and age differences (girls more active than boys, and older adolescents more active than younger ones) [11,17,19], preponderance of African studies support our findings that girls tend to be less active than boys, and that for both boys and girls there is a consistent physical activity decline with increasing age [6,9,12–14,18,20,45,51]. Moreover, our findings provide extra insights into the patterns of physical activity behaviours between girls and boys. While Nigerian adolescent girls would spend more time than boys in domestic and light-intensity physical activities, boys would spend more time in leisure-time and vigorous-intensity activities than girls. These distinct behavioural patterns of physical activity between girls and boys confirm the potential influence of social orientation on adolescents’ health behaviours in Africa and highlight important opportunities for gender specific interventions for physical activity promotion among Nigerian adolescents.

Direct comparison of the present study findings on differences in physical activity by adolescents’ body weight status with results from other African studies may not be made because many of these studies have used continuous BMI variable as a proxy of weight status [13–15,20,45,46]. In contrast to some studies from western high income countries showing underweight adolescents were less likely to be physically active [22,54], underweight adolescents in our study were more active at leisure-time than their normal weight and obese counterparts, engaged in more moderate-intensity activity than overweight adolescents, and accumulated more total physical activity than obese adolescents. However, consistent with other studies showing differences in physical activity between normal weight and overweight adolescents [44,55], normal weight adolescents engaged in more moderate-intensity physical activity than overweight adolescents, and their total physical activity levels were higher than those for the obese adolescents. Discrepant findings or lack of consistency among studies on the relationships between weight status and physical activity could suggest that cross-sectional associations of adolescents’ physical activity with body weight status may not represent much information and caution should be exercised in interpreting or drawing conclusions from such relationships. Perhaps, the use of prospective longitudinal design by future Africa studies could better help explain the potential associations of physical activity patterns and body weight status in African adolescents.

Consistent with the results from other studies [5,45,46,56], our findings indicate that higher family SES was associated with more leisure-time physical activity and moderate-to vigorous intensity activity but with less active transportation. African adolescents from high SES family may have more financial leverage to engage in leisure time sporting activities and use motorized transportation to school compared to adolescents from low SES families who may have fewer opportunities to participate in formal sports but walk more out of no choice than their privileged peers [16]. However, we acknowledge the fact that only few African studies have examined the influence of SES on the various domains of physical activity among adolescents [45,46], and that the relationship between adolescents’ physical activity and SES could be more complex than as espoused in our study [57]. Thus, it may be early to assume that lower SES is primarily associated with reduced moderate to vigorous physical activity among Nigerian adolescents.

There are strengths and limitations to this study. The primary strength is the focus on urban African adolescents’ which is a population at an increased risk of development of physical inactivity related NCDs that have been linked to the rapid urbanization and ongoing physical activity transition in Africa [14,15,58,59]. Other strengths of this study include the examination of demographic-specific associations using a valid and established instrument that allowed the identification of domains and intensity levels of physical activity that could be important for different adolescents’ subgroups. Also, the large representative sample of secondary school adolescents could enhance the generalization of results to this population. However, stemming from the primary strength of the study is also a limitation. The focus on urban population of secondary school students could produce a sample that is high in SES (education) and this may not generalize to the general adolescents’ population in Nigeria. Current estimates indicate that only about 44% (41% female; 47% male) of the 53.5 million young people aged 10–24 years in Nigeria were enrolled in secondary schools [31]. However, understanding the contexts of physical activity during the high school years is important to public health and can be helpful in designing interventions during adolescence [23]. Other limitations include the cross-sectional nature of the study that makes it difficult to draw firm conclusions of causality on the associations found. Also, the use of self-report measure might have introduced some measurement bias, recall problems and inaccurate estimates of physical activity intensities. Objective measures of physical activity such as accelerometers or pedometers provide better estimates of total physical activity of different intensities. However, accelerometers or pedometers do not provide information on the type of activity done. From a public health perspective, it is important to know the contexts in which physical activity occur in adolescents [21,22]. Moreover, contextual data as reported in the present study provides a more comprehensive understanding of specific types of physical activity behaviours and robust opportunity for domain-specific modelled interventions [23,60].

In conclusion, the present study showed that most secondary school adolescents in Nigeria were not meeting the international recommendations for sufficient physical activity, and that school-based physical activity and light intensity activity were the most prominent domain and intensity-category of self-reported physical activity, respectively in these adolescents. Younger age, boys, high family SES and household car ownership were associated with more leisure-time physical activity, moderate-to-vigorous physical activity and total physical activity, while high family SES and household car ownerships were associated with reduced active transportation to and from school. These findings provide information that could be useful for surveillance and public health planning in Nigeria, and have implications for identifying the contexts where adolescents’ physical activity could best be promoted and the subgroups that should be targeted for effective physical activity promoting interventions.

## Supporting Information

S1 Appendix Anonymized Dataset. (PDF)
