# Supplementary material for: Patterns and Associated Factors of Physical Activity among Adolescents in Nigeria
Source: PLoS One. 2016 Feb 22;11(2):e0150142. doi: 10.1371/journal.pone.0150142 (PMC4762937; doi:10.1371/journal.pone.0150142)
Supplement: S1 Appendix — (PDF) [file pone.0150142.s001.pdf]

Anonymized data of the 1006 participants

No	Grade	SES	BMI	School PA	House PA	Leisure PA	AT	LPA	MPA	VPA	MVPA	TPA
1	JSS 2	Low	15.24	1520	280	0	150	1305	90	105	195	1875
2	JSS 3	Low	16.56	1600	455	90	100	1235	470	50	520	2195
3	JSS 3	Low	15.52	1707	1680	210	400	2100	637	270	907	3797
4	SS 2	Low	17.85	1800	1680	30	0	2100	1260	870	2130	3510
5	JSS 3	Low	15.35	1800	1260	0	100	1620	720	470	1190	3110
6	JSS 2	Low	16.41	1800	150	20	0	1300	375	75	450	1970
7	SS 1	Low	17.51	1250	150	0	0	1010	150	0	150	1400
8	SS 1	Low	17.3	1500	75	0	0	1200	70	5	75	1575
9	SS 1	Low	14.18	1600	100	20	0	1225	175	0	175	1720
10	SS 3	Low	15.41	1635	330	0	200	1290	240	135	375	2075
11	SS 1	Low	17.63	1580	390	180	0	1410	30	230	260	2150
12	JSS 2	Low	17.22	565	1680	0	350	1240	435	510	945	2395
13	SS 1	Low	16.02	1525	900	0	0	1620	235	270	505	2425
14	JSS 3	Low	15.87	1500	690	0	600	1680	210	0	210	2490
15	SS 2	Low	17.01	985	1485	35	0	825	1220	425	1645	2505
16	JSS 3	Low	17.78	1670	240	105	300	1320	90	200	290	2165
17	JSS 2	Low	18.03	1660	980	0	0	1620	430	290	720	2640
18	JSS 3	Low	19.78	1270	277	0	100	1277	150	120	270	1597
19	SS 1	Low	19.61	1500	150	120	400	1200	0	150	150	1970
20	JSS 3	Low	23.31	1580	525	0	0	1235	230	340	570	2105
21	JSS 2	Low	20	1510	550	20	0	1620	20	120	140	2080
22	JSS 3	Low	18.49	1650	480	0	300	1410	210	210	420	2280
23	JSS 3	Low	19.84	1650	570	90	0	1620	300	0	300	2310
24	JSS 2	Low	21.36	1100	1275	0	0	1400	525	450	975	2375
25	JSS 3	Low	18.08	1600	875	60	100	1235	470	470	940	2585
26	JSS 3	Low	20.34	1600	875	120	100	1235	470	470	940	2645
27	SS 1	Low	20.43	1500	1200	0	0	1620	420	360	780	2700
28	JSS 3	Low	19.29	1690	1330	0	0	1830	460	430	890	3020
29	JSS 3	Low	20.03	1800	1470	0	100	2100	720	175	895	3320
30	JSS 2	Low	25.39	1680	1500	90	0	2100	390	30	420	3270
31	JSS 2	Low	27.12	1655	1620	20	300	2040	425	510	935	3445
32	SS 1	Low	18.49	1500	1680	150	200	2100	280	840	1120	3430
33	JSS 2	Low	18.26	1800	1485	0	95	1795	885	465	1350	3485
34	SS 1	Low	24.91	1575	1680	270	0	2100	700	915	1615	3525
35	SS 1	Low	18.52	960	150	70	0	935	70	105	175	1180
36	JSS 3	Low	18.55	1515	205	50	150	1235	40	145	185	1845
37	JSS 3	Low	17.92	1775	980	225	0	1690	615	150	765	2980
38	JSS 2	Low	27.14	0	255	0	250	150	900	105	1005	345
39	JSS 2	Low	18.49	1575	65	0	0	1225	40	75	115	1640

40	JSS 3	Low	21.75	1270	385	0	250	1235	220	200	420	1705
41	SS 3	Low	24.49	950	670	0	300	1020	120	480	600	1770
42	SS 3	Low	25.16	1500	300	120	0	1200	300	0	300	1920
43	SS 1	Low	18.18	1500	310	10	0	1235	35	240	275	1820
44	SS 3	Low	18.73	1520	140	0	600	1340	20	0	20	1960
45	JSS 2	Low	18.22	1510	357	70	150	1347	80	140	220	2012
46	SS 1	Low	22.06	1500	630	0	100	1620	0	210	210	2180
47	JSS 3	Low	20.7	1540	455	180	300	1320	225	150	375	2325
48	SS 1	Low	20.03	1800	319	0	600	1249	30	540	570	2419
49	JSS 2	Low	18.22	1540	870	5	120	1440	210	460	670	2475
50	JSS 3	Low	18.44	1600	875	120	100	1235	470	470	940	2645
51	JSS 3	Low	18.97	1650	1050	120	0	1620	210	570	780	2820
52	JSS 3	Low	19.15	1630	470	0	100	1235	430	135	565	2150
53	JSS 3	Low	22.19	1600	1295	120	100	1235	470	890	1360	3065
54	SS 1	Low	18.52	1500	1380	60	360	2040	420	120	540	3120
55	JSS 3	Low	19.72	1800	1170	0	300	1920	510	240	750	3120
56	SS 2	Low	20.2	1575	1435	90	0	1725	960	25	985	3100
57	SS 3	Low	21.22	1525	1085	50	100	2100	60	0	60	2710
58	SS 2	Low	24.16	1500	1680	180	0	2040	280	840	1120	3360
59	SS 1	Low	28.37	1550	1204	315	300	2040	280	134	414	3219
60	JSS 2	Low	17.24	300	0	0	0	250	0	50	50	300
61	JSS 2	Low	30.67	180	500	30	0	375	170	135	305	710
62	JSS 2	Low	36.65	1460	1680	60	0	2100	430	410	840	3200
63	JSS 3	Low	15.11	1700	210	180	250	1305	255	50	305	2215
64	JSS 2	Low	15.82	1600	445	180	0	1620	75	50	125	2225
65	JSS 2	Low	16.23	1550	570	30	0	1620	200	0	200	2150
66	JSS 3	Low	16.4	1600	595	120	100	1235	470	190	660	2365
67	JSS 2	Low	17.26	1605	434	30	100	1270	129	340	469	2119
68	JSS 3	Low	16.44	1680	900	0	0	1620	90	570	660	2580
69	JSS 2	Low	17.85	1575	904	120	100	2054	75	50	125	2649
70	JSS 2	Low	16.16	1560	960	0	150	1560	190	470	660	2595
71	JSS 3	Low	17.31	1570	105	0	0	1235	55	85	140	1675
72	JSS 3	Low	17.86	1630	125	0	100	1235	80	140	220	1805
73	JSS 3	Low	17.47	1515	385	0	100	1375	140	85	225	1950
74	JSS 3	Low	16.42	1535	173	240	100	1275	58	75	133	1998
75	JSS 2	Low	16.02	1510	420	70	100	1340	220	70	290	2050
76	JSS 3	Low	17.4	1670	125	420	300	1235	90	170	260	2365
77	JSS 2	Low	17.9	1510	455	140	100	1235	220	210	430	2155
78	JSS 3	Low	16.23	1600	1295	120	100	1235	470	890	1360	3065
79	JSS 2	Low	16.88	1610	1680	0	0	2100	100	460	560	3290
80	JSS 2	Low	16.9	1660	1115	300	300	1235	850	390	1240	3225
81	SS 1	Low	16.36	1200	490	0	0	1320	335	35	370	1690
82	JSS 2	Low	17.4	1800	75	55	0	1230	25	55	80	1930

83	SS 1	Low	12.82	1800	290	0	100	1340	420	130	550	2140
84	SS 1	Low	16.9	1700	760	10	0	1480	620	60	680	2470
85	SS 1	Low	15.61	1850	540	210	0	1560	230	300	530	2600
86	JSS 2	Low	14.1	1660	400	75	0	1410	80	270	350	2135
87	SS 2	Low	17.9	1560	1680	210	0	2100	870	180	1050	3450
88	JSS 3	Low	20.28	1265	245	0	100	1235	75	200	275	1560
89	JSS 3	Low	16.18	1515	0	10	0	1200	15	0	15	1525
90	JSS 2	Low	16	1550	115	0	0	1280	15	70	85	1665
91	JSS 2	Low	15.11	1600	10	20	0	1200	50	60	110	1630
92	JSS 2	Low	17.15	1550	220	0	0	1290	70	110	180	1770
93	JSS 2	Low	15.43	1550	84	180	0	1249	35	50	85	1814
94	JSS 3	Low	17.72	1600	490	120	100	1235	470	85	555	2260
95	SS 3	Low	15.94	1500	490	0	600	1620	70	0	70	2290
96	JSS 2	Low	13.22	1510	945	30	150	1270	45	840	885	2560
97	JSS 3	Low	16.2	1800	120	90	100	1235	370	40	410	2060
98	JSS 3	Low	14.86	1660	870	30	0	1560	100	570	670	2560
99	JSS 3	Low	15.94	1570	490	225	230	1305	160	295	455	2425
100	JSS 2	Low	16.73	1510	410	160	100	1260	220	140	360	2130
101	JSS 3	Low	16.66	1600	1050	60	200	2040	0	310	310	2810
102	JSS 3	Low	16.23	1630	1260	0	100	1620	450	520	970	2940
103	SS 1	Low	17.57	450	1680	0	0	1240	680	840	1520	2130
104	SS 1	Low	19.29	1500	35	0	0	1235	0	0	0	1535
105	JSS 2	Low	19.78	1550	190	30	0	1290	150	0	150	1770
106	JSS 2	Low	25.44	1650	260	120	0	1320	140	150	290	2030
107	JSS 2	Low	21.37	1800	240	90	0	1410	20	610	630	2130
108	SS 3	Low	27.06	1575	1505	210	200	2100	85	235	320	3390
109	JSS 2	Low	18.55	1655	1120	420	0	1620	425	430	855	3195
110	JSS 2	Low	20.55	1800	1500	10	150	2100	1170	180	1350	3385
111	JSS 2	Low	20.82	1655	0	210	150	1200	635	420	1055	1940
112	JSS 3	Low	18.18	1210	175	0	100	1235	75	75	150	1415
113	SS 1	Low	17.86	1575	85	0	0	1235	85	40	125	1660
114	JSS 3	Low	27.25	1700	105	0	0	1200	255	50	305	1805
115	JSS 3	Low	17.78	1505	180	0	100	1340	25	20	45	1735
116	JSS 2	Low	20.2	1575	185	20	100	1350	75	35	110	1830
117	JSS 3	Low	20.96	1560	300	0	0	1340	150	70	220	1860
118	JSS 3	Low	19.29	1570	345	0	100	1235	220	160	380	1965
119	SS 1	Low	21.21	1550	525	0	120	1410	155	210	365	2135
120	JSS 3	Low	20.03	1600	490	120	100	1235	470	85	555	2260
121	JSS 3	Low	20.44	1585	520	30	300	1410	220	175	395	2285
122	JSS 3	Low	18.22	1600	595	120	100	1235	470	190	660	2365
123	SS 1	Low	20.69	1700	540	20	250	1290	470	180	650	2385
124	JSS 2	Low	19.63	1575	410	250	100	1225	75	385	460	2285
125	SS 1	Low	20.43	1650	910	5	0	2040	120	100	220	2565

126	JSS 3	Low	22.49	1800	710	0	120	1620	450	165	615	2570
127	JSS 2	Low	18.07	1510	735	100	300	1725	40	180	220	2495
128	JSS 3	Low	20.61	1600	875	180	100	1235	470	470	940	2705
129	JSS 2	Low	20.78	1520	1085	60	0	2110	45	150	195	2665
130	JSS 2	Low	20.03	1700	375	180	600	1275	200	300	500	2705
131	SS 1	Low	18.37	1700	510	60	0	1260	470	180	650	2270
132	JSS 2	Low	19.26	1800	1260	0	0	1620	570	570	1140	3060
133	SS 1	Low	18.47	1550	1680	60	0	2100	840	190	1030	3290
134	JSS 2	Low	22.03	1705	1680	0	0	2100	285	620	905	3385
135	JSS 3	Low	20.31	1500	1680	0	0	2100	910	350	1260	3180
136	JSS 2	Low	18.52	1655	1150	270	900	1360	635	510	1145	3625
137	SS 1	Low	23.5	1650	1680	70	300	2010	1140	1020	2160	3550
138	JSS 3	Low	21.52	1265	105	0	50	1235	75	60	135	1420
139	JSS 2	Low	18.26	1510	119	5	0	1249	45	35	80	1634
140	JSS 2	Low	21.26	1525	15	5	300	1215	0	25	25	1695
141	SS 1	Low	18.43	775	1020	0	0	1020	570	205	775	1795
142	JSS 3	Low	21.76	1600	455	30	100	1235	470	50	520	2135
143	SS 2	Low	23.24	1500	1290	30	400	2040	210	240	450	3020
144	JSS 2	Low	24.77	1580	931	70	0	2040	75	96	171	2581
145	SS 1	Low	19.68	1800	1580	30	720	2100	730	40	770	3770
146	SS 3	Low	19.37	1550	1680	300	150	2100	25	865	890	3605
147	SS 2	Low	18.59	1630	1680	105	0	2040	920	200	1120	3415
148	SS 1	Low	23.88	1800	1680	315	0	2100	1260	450	1710	3780
149	SS 3	Low	19.33	390	390	0	0	540	240	0	240	780
150	SS 1	Low	18.55	600	105	0	300	515	70	0	70	855
151	SS 1	Low	19.47	1260	179	5	0	1009	70	120	190	1444
152	JSS 3	Low	23.5	1270	245	0	100	1235	80	200	280	1565
153	JSS 3	Low	18.97	1590	0	0	100	1200	30	60	90	1640
154	SS 2	Low	17.92	1560	0	40	120	1200	60	0	60	1660
155	SS 1	Low	21.22	1600	105	28	0	1235	70	100	170	1733
156	JSS 2	Low	22.86	1510	235	15	0	1270	150	25	175	1760
157	JSS 2	Low	21.48	1550	300	0	0	1320	60	170	230	1850
158	JSS 2	Low	20.06	1525	385	0	0	1270	105	235	340	1910
159	SS 1	Low	26.49	1500	420	0	0	1620	0	0	0	1920
160	JSS 3	Low	19.61	1500	430	0	0	1560	70	0	70	1930
161	JSS 3	Low	19.29	1520	385	0	150	1235	220	150	370	1980
162	JSS 2	Low	18.13	1525	360	15	0	1560	0	25	25	1900
163	JSS 3	Low	18.36	1800	240	0	100	1230	330	205	535	2090
164	JSS 2	Low	20.06	1550	505	45	100	1440	75	240	315	2150
165	SS 1	Low	18.73	1700	510	10	0	1260	470	180	650	2220
166	JSS 3	Low	24.3	1650	350	60	400	1410	0	290	290	2260
167	JSS 3	Low	16.82	1625	590	120	250	1620	140	155	295	2460
168	SS 1	Low	18.56	500	1500	30	0	1290	470	240	710	2030

169	JSS 3	Low	17.91	1800	355	0	120	1500	625	80	705	2215
170	SS 3	Low	18.37	1500	980	0	400	2040	140	0	140	2680
171	SS 2	Low	26.37	1600	930	210	0	1620	210	400	610	2740
172	JSS 2	Low	19.23	1655	260	210	100	1320	145	150	295	2175
173	SS 3	Low	18.73	1775	1050	0	0	1410	360	755	1115	2825
174	SS 1	Low	23.25	1550	1302	0	120	1242	35	1275	1310	2912
175	SS 2	Low	18.2	1500	1360	60	0	2040	280	240	520	2920
176	SS 1	Low	19.38	1800	840	0	600	2040	40	300	340	2940
177	JSS 3	Low	22.01	1550	1295	120	100	1235	470	840	1310	3015
178	SS 2	Low	23.67	1500	1410	0	300	2040	420	150	570	3060
179	JSS 3	Low	18.52	1800	1210	0	100	2040	370	325	695	3040
180	JSS 3	Low	22.89	1600	1295	120	200	1235	470	890	1360	3065
181	SS 3	Low	21.61	1500	1160	60	300	2110	250	0	250	2870
182	SS 1	Low	18.86	1500	1680	0	0	2040	840	100	940	3180
183	SS 3	Low	21.83	1500	1260	0	900	2040	420	0	420	3360
184	JSS 3	Low	18	1800	1220	0	0	1760	750	660	1410	3020
185	SS 3	Low	22.48	1500	900	315	0	1800	180	120	300	2715
186	SS 1	Low	21.1	1500	1680	0	0	2040	840	180	1020	3180
187	SS 1	Low	18.42	1700	1680	150	200	2100	280	1040	1320	3630
188	JSS 2	Low	30.86	390	240	20	0	280	260	90	350	650
189	JSS 3	Low	28.96	1650	840	30	0	2040	0	150	150	2520
190	SS 3	Low	29.39	1500	840	25	300	2040	0	0	0	2515
191	SS 1	Low	30.41	1500	1358	60	150	1620	469	469	938	2993
192	JSS 2	Low	33.91	945	1225	0	0	2100	320	50	370	2170
193	JSS 2	Low	39.04	1650	430	0	300	1340	210	230	440	2230
194	JSS 2	Medium	15.11	1530	735	60	100	1270	660	35	695	2375
195	JSS 2	Medium	17.07	1510	1680	90	0	1680	850	420	1270	3280
196	JSS 3	Medium	17.01	1570	120	0	200	1235	80	75	155	1790
197	JSS 3	Medium	16.44	1625	385	0	100	1235	275	200	475	2060
198	JSS 2	Medium	15.38	1695	600	150	150	1725	55	215	270	2520
199	JSS 3	Medium	15.56	1595	260	315	200	1340	20	195	215	2270
200	JSS 3	Medium	16.41	1800	1680	0	120	1620	1260	445	1705	3540
201	SS 3	Medium	16.45	1550	230	0	0	1400	80	0	80	1780
202	JSS 3	Medium	16.89	1825	65	0	200	1235	300	55	355	1920
203	JSS 3	Medium	17.69	1570	385	0	100	1235	220	200	420	2005
204	SS 3	Medium	16.33	1410	630	0	200	1410	270	210	480	2140
205	JSS 3	Medium	16.03	1800	700	0	100	1620	440	170	610	2550
206	JSS 3	Medium	15.05	1650	710	300	0	1550	150	360	510	2660
207	JSS 3	Medium	19.63	1265	315	0	100	1235	215	130	345	1630
208	JSS 3	Medium	19.5	1690	285	0	100	1235	80	360	440	2025
209	JSS 3	Medium	22.31	1600	405	0	200	1410	105	190	295	2105
210	JSS 3	Medium	19.43	1570	525	0	100	1235	220	340	560	2145
211	JSS 3	Medium	18.82	1265	105	0	250	1235	40	95	135	1420

212	JSS 3	Medium	19.43	1570	525	180	100	1235	220	340	560	2325
213	SS 2	Medium	21.51	185	420	0	0	360	215	30	245	605
214	JSS 3	Medium	19.98	1270	315	0	100	1235	220	130	350	1635
215	JSS 3	Medium	18.03	1550	85	0	100	1225	60	50	110	1685
216	SS 1	Medium	25.1	1660	120	0	0	1320	10	150	160	1780
217	JSS 3	Medium	20.45	1265	315	180	100	1235	215	130	345	1810
218	SS 3	Medium	19.72	1550	320	0	0	1400	170	0	170	1870
219	JSS 3	Medium	18.31	1570	280	0	100	1340	80	130	210	1900
220	JSS 3	Medium	20.45	1525	315	0	150	1249	148	143	291	1915
221	JSS 3	Medium	24.52	1525	630	0	0	1620	210	25	235	2155
222	JSS 3	Medium	18.66	1570	525	180	100	1235	220	340	560	2325
223	JSS 3	Medium	18.67	1600	665	180	100	1235	470	260	730	2495
224	JSS 3	Medium	22.31	1800	546	0	125	1235	720	141	861	2406
225	SS 3	Medium	17.99	1740	220	300	100	1230	250	180	430	2360
226	JSS 3	Medium	23.14	1505	1260	0	0	1410	215	840	1055	2765
227	JSS 3	Medium	19.47	1590	590	180	150	1240	85	555	640	2435
228	JSS 3	Medium	20.5	1800	1010	0	100	1220	1140	175	1315	2860
229	SS 3	Medium	16.53	1525	1260	180	150	1620	865	0	865	3040
230	SS 3	Medium	21.26	1500	1680	0	0	2100	210	480	690	3180
231	SS 1	Medium	24.52	1510	1575	0	0	1620	325	840	1165	3085
232	JSS 2	Medium	21.41	1510	1410	150	0	1830	430	360	790	3070
233	JSS 2	Medium	18.29	1505	1470	210	400	1830	425	420	845	3385
234	JSS 2	Medium	20.69	1505	1710	210	0	1830	425	660	1085	3425
235	JSS 2	Medium	26.04	1510	1680	350	400	1830	430	770	1200	3740
236	JSS 2	Medium	19.68	1625	1414	420	150	2100	145	134	279	3534
237	JSS 2	Medium	14.88	1510	70	90	0	1225	20	35	55	1670
238	JSS 3	Medium	14.95	1800	0	0	200	1200	300	50	350	1860
239	SS 1	Medium	16.18	1500	35	175	0	1235	0	0	0	1710
240	JSS 2	Medium	17.09	1530	140	240	0	1220	125	25	150	1910
241	JSS 3	Medium	15.43	1700	515	10	0	1340	515	60	575	2225
242	JSS 2	Medium	17.8	1700	540	0	200	1560	260	120	380	2340
243	JSS 3	Medium	17.8	1530	320	120	0	1380	75	95	170	1970
244	JSS 3	Medium	16.85	1265	255	0	100	1235	215	70	285	1570
245	JSS 3	Medium	17.26	1525	70	0	100	1235	35	325	360	1645
246	JSS 3	Medium	17.05	1270	315	0	150	1235	80	270	350	1655
247	JSS 3	Medium	16.02	1270	315	0	150	1235	220	130	350	1660
248	JSS 3	Medium	13.89	1650	460	0	0	1410	210	190	400	2110
249	JSS 3	Medium	17.6	1600	595	120	100	1235	470	190	660	2365
250	JSS 3	Medium	17.21	1560	900	15	100	2040	45	75	120	2525
251	JSS 3	Medium	17.6	1630	525	45	150	1235	280	340	620	2225
252	JSS 2	Medium	13.24	1600	530	180	0	1310	340	180	520	2310
253	JSS 3	Medium	16.4	1600	1078	0	100	2075	110	193	303	2728
254	JSS 3	Medium	16.44	1595	1190	40	0	1830	335	320	655	2825

255	JSS 2	Medium	17.33	1520	1120	180	0	1690	230	420	650	2820
256	JSS 2	Medium	17.31	1655	1155	20	0	1830	425	255	680	2830
257	SS 1	Medium	17.54	1555	115	0	0	1270	75	25	100	1670
258	SS 1	Medium	17.36	1500	110	210	0	1210	0	100	100	1820
259	JSS 3	Medium	15.24	1560	420	90	0	1340	150	190	340	2070
260	JSS 2	Medium	16.63	1575	305	150	100	1410	75	95	170	2080
261	JSS 3	Medium	15.63	1535	240	245	0	1270	160	45	205	2020
262	JSS 3	Medium	17.54	1800	1050	420	100	1620	735	460	1195	3320
263	SS 2	Medium	17.36	95	290	180	200	195	160	30	190	665
264	JSS 3	Medium	16.63	1265	140	0	100	1235	40	130	170	1455
265	SS 3	Medium	17.36	880	540	100	300	1080	240	100	340	1670
266	SS 3	Medium	16.53	1510	210	0	300	1340	80	0	80	1870
267	JSS 3	Medium	17.4	1575	140	180	100	1235	85	95	180	1945
268	JSS 2	Medium	16.44	1510	660	120	0	1620	115	135	250	2290
269	SS 3	Medium	17.58	1545	300	20	300	1260	195	90	285	2015
270	JSS 3	Medium	17.48	1600	560	120	0	1270	470	120	590	2280
271	JSS 3	Medium	17.1	1800	490	0	120	1235	335	445	780	2350
272	JSS 3	Medium	19.07	1590	420	420	0	1340	180	190	370	2430
273	SS 3	Medium	17.36	1500	900	36	0	2100	0	0	0	2436
274	JSS 2	Medium	16.89	1505	700	210	0	1620	215	70	285	2415
275	JSS 3	Medium	14.61	1800	1680	0	100	1620	720	1165	1885	3530
276	JSS 3	Medium	21.63	1265	175	0	100	1235	75	130	205	1490
277	JSS 2	Medium	21.76	1500	10	210	0	1210	0	0	0	1720
278	SS 3	Medium	23.59	1675	390	0	0	1560	150	55	205	2065
279	JSS 3	Medium	18.85	1600	525	120	100	1235	120	470	590	2295
280	JSS 3	Medium	18.52	1585	840	0	0	1560	310	255	565	2425
281	JSS 2	Medium	19.61	1695	640	300	200	1620	210	205	415	2735
282	JSS 3	Medium	20.72	1610	1680	0	200	1620	430	940	1370	3390
283	SS 1	Medium	16.02	1800	1025	0	0	2040	815	345	1160	2825
284	JSS 2	Medium	20.25	1654	1577	45	100	2100	221	250	471	3276
285	JSS 3	Medium	19.92	1270	265	0	100	1235	220	80	300	1585
286	JSS 3	Medium	22.15	1270	265	0	150	1235	220	80	300	1610
287	JSS 3	Medium	18.02	1500	100	60	100	1250	50	0	50	1710
288	JSS 3	Medium	19.9	1610	220	0	100	1305	120	105	225	1880
289	JSS 3	Medium	18.92	1650	210	60	300	1410	0	150	150	2070
290	JSS 2	Medium	19	1510	465	280	0	1245	220	210	430	2255
291	JSS 2	Medium	22.95	1550	840	180	0	1200	890	0	890	2570
292	JSS 3	Medium	19.07	1600	875	60	100	1235	470	470	940	2585
293	SS 1	Medium	19.1	1625	1070	0	0	1550	470	375	845	2695
294	JSS 2	Medium	21.63	1575	979	90	100	2054	75	125	200	2694
295	JSS 2	Medium	21.78	1510	990	200	100	1270	370	560	930	2750
296	JSS 3	Medium	18.26	1585	1260	180	0	1830	430	285	715	3025
297	JSS 3	Medium	19.13	1800	1680	100	100	2040	440	720	1160	3630

298	SS 2	Medium	21.8	505	260	0	0	540	120	105	225	765
299	JSS 2	Medium	18.55	55	210	0	75	220	40	155	195	340
300	JSS 3	Medium	18.03	1800	147	0	100	1242	359	56	415	1997
301	JSS 2	Medium	18.87	1535	300	70	0	1410	80	45	125	1905
302	SS 3	Medium	21.79	1550	750	0	0	1800	200	0	200	2300
303	JSS 3	Medium	19.95	1550	780	15	0	1920	35	75	110	2345
304	SS 2	Medium	19.61	1320	1050	0	75	1590	220	320	540	2520
305	JSS 3	Medium	20.31	1680	965	30	0	1620	155	570	725	2675
306	JSS 2	Medium	22.07	1530	610	40	0	1410	365	65	430	2180
307	SS 1	Medium	18.37	1500	1285	210	300	2100	0	25	25	3145
308	JSS 3	Medium	22.6	1800	980	140	100	1620	765	440	1205	2970
309	JSS 2	Medium	20.28	1560	1285	30	0	1655	570	320	890	2875
310	JSS 2	Medium	21.77	1500	1220	140	0	1760	420	240	660	2860
311	JSS 3	Medium	22.23	1670	1680	0	300	2100	650	330	980	3500
312	JSS 3	Medium	20.08	1800	1680	0	100	2040	720	1020	1740	3530
313	JSS 2	Medium	21.3	300	315	0	0	320	35	260	295	615
314	SS 1	Medium	23.14	1500	0	60	0	1200	0	0	0	1560
315	JSS 3	Medium	19.6	1265	490	0	100	1235	40	480	520	1805
316	SS 1	Medium	20.96	400	840	0	600	820	420	0	420	1840
317	JSS 3	Medium	21.79	1565	175	45	100	1235	75	130	205	1835
318	JSS 2	Medium	17.99	1550	210	0	300	1235	70	155	225	1910
319	SS 3	Medium	18.04	1250	529	0	300	1620	120	39	159	1929
320	JSS 3	Medium	20.2	1200	0	210	0	1200	0	0	0	1410
321	JSS 3	Medium	21.83	1800	260	20	0	1260	40	460	500	2080
322	JSS 3	Medium	23.11	1265	889	0	100	1249	5	900	905	2204
323	SS 1	Medium	23.11	1500	750	0	0	1620	210	120	330	2250
324	JSS 3	Medium	21.05	1570	700	0	100	1270	220	480	700	2320
325	JSS 3	Medium	19.22	1570	525	180	100	1235	220	340	560	2325
326	SS 2	Medium	19	1600	640	140	0	1760	60	120	180	2380
327	JSS 2	Medium	18.43	375	1680	120	0	1485	260	520	780	2175
328	SS 3	Medium	20.81	1775	630	0	150	1410	360	335	695	2480
329	JSS 3	Medium	17.36	1725	555	210	0	1410	390	180	570	2490
330	JSS 2	Medium	21.01	1560	900	90	0	1410	220	530	750	2550
331	JSS 3	Medium	17.58	1800	700	0	100	1270	720	235	955	2550
332	JSS 3	Medium	18.73	1800	700	0	120	1270	720	235	955	2560
333	JSS 3	Medium	19.2	1565	575	75	0	1440	225	175	400	2215
334	JSS 2	Medium	19.53	1505	762	0	0	1500	462	5	467	2267
335	JSS 3	Medium	18.9	1800	1050	0	100	1620	720	260	980	2900
336	SS 3	Medium	20.03	1775	1050	0	300	1410	360	755	1115	2975
337	JSS 3	Medium	20.82	1570	1085	50	100	1235	220	900	1120	2755
338	JSS 3	Medium	18.13	1595	1470	0	0	2100	210	95	305	3065
339	SS 2	Medium	20.2	1690	1170	30	400	1650	490	420	910	3090
340	JSS 3	Medium	18.87	1675	1456	0	100	1270	1410	151	1561	3181

341	SS 1	Medium	20.18	1550	995	0	230	1235	840	170	1010	2675
342	JSS 3	Medium	22.48	1800	1330	0	100	1620	1140	120	1260	3180
343	JSS 3	Medium	17.94	1800	1358	0	120	2100	363	85	448	3218
344	SS 2	Medium	18.51	1800	1260	210	0	2040	290	460	750	3270
345	JSS 3	Medium	26.3	1770	1540	120	0	2100	265	495	760	3430
346	JSS 2	Medium	23.05	1550	750	50	900	1560	200	240	440	3250
347	SS 2	Medium	22.86	1410	1680	150	0	2220	510	330	840	3240
348	JSS 2	Medium	21.97	1500	1050	300	20	1830	420	0	420	2860
349	JSS 2	Medium	18.9	1500	1160	210	0	1760	420	180	600	2870
350	SS 2	Medium	26.37	1800	1505	120	150	1515	1030	725	1755	3500
351	SS 2	Medium	20.44	1800	1260	0	0	2100	450	450	900	3060
352	SS 3	Medium	21.67	1800	1680	120	300	1830	720	1040	1760	3750
353	SS 1	Medium	26.62	1550	1680	105	300	1935	560	470	1030	3485
354	SS 1	Medium	21.78	1700	1680	150	200	2100	280	1040	1320	3630
355	JSS 3	Medium	27.78	1625	455	0	100	1235	275	270	545	2130
356	SS 2	Medium	28.58	1500	1309	0	150	1620	420	469	889	2884
357	SS 2	Medium	18.97	1800	1260	60	0	1620	940	300	1240	3120
358	SS 1	Medium	16.59	775	735	0	0	1020	360	130	490	1510
359	SS 2	Medium	27.06	1500	395	0	0	1340	210	45	255	1895
360	JSS 3	High	14.81	1575	875	0	100	1235	470	445	915	2500
361	JSS 3	High	16.44	1800	700	0	100	1270	720	235	955	2550
362	JSS 2	High	14.57	1205	280	20	0	725	500	260	760	1505
363	SS 1	High	19.63	1575	0	5	0	1200	50	25	75	1580
364	SS 1	High	23.46	1500	238	0	0	1340	70	28	98	1738
365	JSS 2	High	18.02	1080	888	0	0	1830	210	228	438	1968
366	JSS 3	High	18.99	1535	285	120	100	1340	80	100	180	1990
367	SS 1	High	17.93	1500	50	120	0	1200	0	50	50	1670
368	SS 1	High	19.07	1650	245	0	0	1410	185	0	185	1895
369	JSS 3	High	23.31	1580	525	300	100	1235	230	340	570	2455
370	SS 2	High	18.2	1685	430	40	0	1410	200	205	405	2155
371	JSS 3	High	27.39	1600	875	120	100	1235	470	470	940	2645
372	JSS 3	High	21.36	1800	515	300	100	1225	370	445	815	2665
373	SS 2	High	18.75	1800	700	40	0	1410	580	510	1090	2540
374	SS 1	High	18.79	1800	1680	90	0	2100	1200	120	1320	3570
375	JSS 3	High	20.45	1800	1365	315	100	2100	370	60	430	3530
376	JSS 3	High	22.52	1570	150	0	100	1235	80	105	185	1770
377	JSS 3	High	19.29	1570	150	0	100	1235	80	105	185	1770
378	JSS 3	High	18.44	1535	315	0	0	1410	80	60	140	1850
379	JSS 3	High	18.82	1560	315	30	0	1270	115	190	305	1905
380	SS 3	High	24.91	0	1680	210	0	1050	420	630	1050	1890
381	SS 2	High	26.67	1500	1680	210	0	2110	420	360	780	3390
382	SS 3	High	20.44	1500	1680	0	600	2100	630	90	720	3480
383	SS 3	High	21.09	850	1200	315	0	1020	510	520	1030	2365

384	SS 2	High	15.82	1500	210	20	0	1410	0	0	0	1730
385	JSS 2	High	13.7	1500	240	60	240	1380	150	210	360	1920
386	JSS 2	High	16.53	1510	720	70	0	1270	640	20	660	2300
387	SS 1	High	16.77	1500	260	140	0	1340	70	50	120	1900
388	JSS 2	High	17.09	1510	1400	210	150	1830	220	560	780	3195
389	JSS 3	High	14.66	1800	854	90	100	1620	720	64	784	2794
390	SS 2	High	16.97	1580	954	90	0	1655	549	30	579	2624
391	JSS 2	High	13.51	1510	275	60	0	1335	80	70	150	1845
392	JSS 3	High	17.36	1575	335	0	100	1340	155	115	270	1960
393	JSS 3	High	17.36	1575	335	0	100	1340	155	115	270	1960
394	JSS 2	High	16.97	1510	360	120	0	1290	220	60	280	1990
395	JSS 3	High	16.89	1600	665	120	100	1235	470	260	730	2435
396	JSS 3	High	13.07	1600	875	60	100	1235	470	470	940	2585
397	JSS 3	High	17.09	1600	1295	120	100	1235	470	890	1360	3065
398	JSS 3	High	16.2	1800	1330	0	100	1270	720	865	1585	3180
399	JSS 2	High	18	1510	1085	70	0	1865	220	210	430	2665
400	JSS 3	High	15.06	1800	1680	0	100	2040	720	445	1165	3530
401	JSS 2	High	17.15	1510	1470	150	150	1410	430	840	1270	3205
402	SS 2	High	15.05	420	670	90	0	370	660	0	660	1180
403	SS 1	High	17.09	1500	85	240	0	1235	0	50	50	1825
404	SS 3	High	17.15	1650	255	0	0	1290	75	240	315	1905
405	JSS 3	High	17.36	1585	265	0	300	1410	40	100	140	2000
406	SS 2	High	17.16	1590	430	210	0	1470	160	90	250	2230
407	JSS 3	High	17.44	1600	670	120	0	1270	470	230	700	2390
408	JSS 3	High	17.35	1646	420	60	250	1200	441	125	566	2251
409	SS 1	High	18.03	1590	1200	210	0	1380	930	180	1110	3000
410	JSS 2	High	16.97	1550	1680	0	0	2100	600	350	950	3230
411	JSS 2	High	15.22	1595	170	180	0	1220	70	175	245	1945
412	JSS 3	High	16.38	1535	250	120	100	1305	80	100	180	1955
413	JSS 2	High	15.94	1530	135	180	0	1260	55	50	105	1845
414	SS 3	High	17.21	1525	710	0	300	1620	165	150	315	2385
415	SS 3	High	16.53	1590	1080	0	0	1620	480	270	750	2670
416	JSS 3	High	16.44	1600	1295	120	100	1235	470	890	1360	3065
417	JSS 3	High	17.67	1650	1245	15	0	1410	315	870	1185	2910
418	JSS 3	High	21.22	1500	70	0	0	1235	35	0	35	1570
419	JSS 2	High	19.15	1575	125	420	125	1290	75	35	110	2170
420	SS 1	High	18.52	1500	98	210	0	1298	0	0	0	1808
421	JSS 2	High	27.01	1600	140	0	300	1270	120	50	170	1890
422	SS 2	High	25.39	1800	70	180	0	1240	180	150	330	2050
423	SS 1	High	19.23	1190	990	10	0	1260	700	220	920	2190
424	JSS 2	High	22.03	1550	630	80	0	1440	200	240	440	2260
425	JSS 2	High	20.45	1655	880	120	0	1270	635	330	965	2655
426	SS 1	High	20.44	1650	1230	210	120	2040	510	330	840	3150

427	SS 2	High	26.4	1580	1470	0	0	1410	920	420	1340	3050
428	SS 1	High	22.1	1800	1020	315	0	1800	1200	120	1320	3135
429	JSS 3	High	22.06	1600	1680	80	0	2100	935	425	1360	3360
430	JSS 2	High	22.27	1530	1290	175	350	1230	845	445	1290	3170
431	JSS 3	High	19.04	1570	1680	30	0	2100	860	470	1330	3280
432	JSS 3	High	22.52	1600	35	120	100	1235	50	50	100	1805
433	JSS 2	High	18.51	1600	200	100	100	1350	75	75	150	1950
434	JSS 2	High	23.05	1530	620	120	0	1340	450	60	510	2270
435	JSS 3	High	18.2	1600	840	180	0	1620	420	100	520	2620
436	SS 1	High	18.07	1800	1005	0	0	1275	1140	140	1280	2805
437	SS 1	High	20.48	1800	870	0	0	1620	1020	105	1125	2670
438	JSS 2	High	23.15	1655	805	350	0	1760	145	255	400	2810
439	JSS 2	High	20.66	1605	780	60	300	1620	215	250	465	2595
440	JSS 3	High	20.55	1800	854	195	100	1620	720	39	759	2899
441	JSS 2	High	23.19	1655	1440	120	300	1620	425	750	1175	3365
442	JSS 3	High	17.94	1540	1170	60	0	1830	100	480	580	2770
443	JSS 3	High	18.31	1575	1680	0	100	1825	725	450	1175	3305
444	SS 3	High	22.04	1500	1680	0	600	2100	630	180	810	3480
445	SS 2	High	24.68	260	305	30	0	290	140	135	275	595
446	SS 1	High	17.78	370	0	75	50	250	0	120	120	495
447	SS 2	High	19.98	1500	35	20	0	1235	0	0	0	1555
448	SS 2	High	20.06	1500	10	70	0	1210	0	0	0	1580
449	SS 1	High	22.13	1500	60	90	0	1235	0	25	25	1650
450	SS 1	High	19.49	1550	20	210	0	1220	50	0	50	1780
451	SS 3	High	18.82	1600	100	20	0	1300	0	100	100	1720
452	SS 2	High	20.83	1610	165	60	0	1290	80	105	185	1835
453	SS 2	High	21.09	775	531	90	0	930	225	151	376	1396
454	JSS 3	High	19.43	1535	287	20	100	1312	80	130	210	1942
455	JSS 2	High	18.82	1550	200	200	0	1225	75	150	225	1950
456	JSS 2	High	26.84	1600	420	0	0	1410	310	0	310	2020
457	SS 1	High	22.15	1600	430	0	300	1360	240	130	370	2045
458	SS 3	High	22.43	1800	340	180	100	1500	190	150	340	2370
459	SS 2	High	27.24	1650	500	120	0	1560	140	150	290	2270
460	SS 2	High	21.34	1590	520	210	0	1620	160	30	190	2320
461	JSS 3	High	22.89	1600	665	120	100	1235	470	260	730	2435
462	SS 2	High	20.44	1670	490	280	0	1410	260	190	450	2440
463	SS 2	High	21.01	1620	850	0	0	1560	150	460	610	2470
464	JSS 3	High	18.29	1800	631	0	150	1250	741	165	906	2481
465	SS 3	High	22.43	1800	380	80	100	1540	190	150	340	2310
466	JSS 3	High	19.48	1600	770	120	100	1270	470	330	800	2540
467	SS 2	High	23.53	1640	424	54	0	1200	214	350	564	2118
468	SS 3	High	19.53	1560	1050	0	0	2040	270	0	270	2610
469	SS 2	High	22.27	1550	420	50	0	1410	235	25	260	2020

470	SS 1	High	20.32	1680	1010	35	0	1305	955	130	1085	2725
471	SS 2	High	22.15	1575	1470	0	0	2040	285	420	705	3045
472	SS 2	High	17.8	1580	455	105	0	1560	65	410	475	2140
473	JSS 3	High	22.68	1800	870	420	100	1230	720	445	1165	3140
474	SS 2	High	21.38	1600	740	175	0	1830	170	40	210	2515
475	SS 2	High	22.49	1730	1470	60	0	1620	920	360	1280	3260
478	JSS 3	High	24.03	1800	595	90	100	1235	720	740	1460	2535
479	JSS 3	High	18.55	1800	960	20	250	1270	350	865	1215	2830
480	JSS 3	High	18.26	1800	525	280	100	1620	370	60	430	2655
481	SS 1	High	20.57	1500	1440	420	0	2100	240	300	540	3360
482	JSS 3	High	20.06	1800	1330	10	100	1620	370	865	1235	3190
483	SS 2	High	21.56	1630	1680	420	60	2100	290	470	760	3760
484	SS 1	High	19.81	1800	1680	180	0	2100	1090	450	1540	3660
485	JSS 3	High	20.28	1290	35	0	150	1235	30	60	90	1400
486	JSS 2	High	24.65	1590	210	20	0	1270	190	40	230	1820
487	JSS 2	High	20.28	1570	110	60	200	1245	65	70	135	1840
488	JSS 3	High	21.08	1700	135	0	100	1235	90	210	300	1885
489	JSS 3	High	20.03	1535	420	0	100	1515	80	60	140	2005
490	JSS 3	High	21.22	1590	135	360	100	1235	80	110	190	2135
491	JSS 2	High	20.7	1550	500	120	0	1560	35	155	190	2170
492	SS 3	High	21.22	1660	350	120	75	1410	120	180	300	2280
493	JSS 2	High	20.31	1650	360	150	100	1410	100	200	300	2210
494	SS 3	High	19.29	1500	920	60	0	1550	210	360	570	2480
495	JSS 3	High	18.02	1800	745	0	100	1225	720	325	1045	2595
496	JSS 3	High	23.68	1800	600	300	100	1500	600	50	650	2750
497	SS 2	High	19.23	1800	450	245	0	1470	300	180	480	2495
498	SS 2	High	22.67	1610	1155	120	0	1935	290	240	530	2885
499	SS 2	High	18.29	1700	1340	120	0	1760	560	420	980	3160
500	SS 1	High	24.24	1800	540	360	300	1350	180	840	1020	2850
501	SS 3	High	21.72	1460	1680	210	0	1680	1065	605	1670	3350
502	JSS 3	High	18.82	1800	1330	60	100	1270	1440	445	1885	3240
503	JSS 3	High	18.03	1800	1680	0	120	1620	930	1230	2160	3540
504	JSS 2	High	28.4	1555	350	10	0	1270	95	240	335	1915
505	JSS 3	High	27.83	1560	1620	0	100	2100	60	360	420	3230
506	SS 2	High	29.38	1700	1680	90	540	1830	920	360	1280	3740
507	JSS 2	High	30.39	1580	515	180	100	1235	65	495	560	2325
508	SS 3	High	28.35	1500	980	0	0	1270	70	840	910	2480
509	SS 2	High	17.21	1640	112	0	0	1235	125	92	217	1752
510	JSS 3	Low	18.26	1500	40	0	0	1210	10	20	30	1540
511	JSS 2	Low	18.02	180	205	360	0	325	40	20	60	745
512	SS 1	Low	15.98	1250	130	150	0	1235	60	85	145	1530
513	JSS 2	Low	13.78	1510	275	90	0	1230	45	210	255	1875
514	SS 1	Low	16.8	1550	129	160	100	1235	95	49	144	1879

515	SS 2	Low	17.75	1560	270	0	100	1250	130	150	280	1880
516	JSS 3	Low	16.77	1515	255	270	0	1200	120	150	270	2040
517	SS 1	Low	30.82	1800	700	40	600	1270	420	510	930	2840
518	SS 2	Low	35.25	1500	1380	0	0	2040	420	120	540	2880
519	SS 2	Low	16.73	1540	980	540	150	1550	420	250	670	3210
520	SS 1	Low	15.24	300	1680	840	100	1510	35	890	925	2870
521	JSS 2	Low	17.26	1550	850	420	900	1270	230	600	830	3470
522	SS 1	Low	16.79	1800	1680	240	300	2040	210	1140	1350	3780
523	SS 2	Low	16.36	1560	160	840	100	1235	150	35	185	2610
524	SS 2	Low	16.54	1560	750	200	0	1410	370	230	600	2510
525	JSS 3	Low	15.43	1600	1650	360	150	2040	630	280	910	3710
526	JSS 2	Low	15.41	1530	45	0	0	1200	30	45	75	1575
527	SS 2	Low	18.29	1590	110	60	0	1250	115	35	150	1760
528	SS 3	Low	18.62	1560	105	240	0	1270	35	60	95	1905
529	JSS 2	Low	16.18	1575	205	180	300	1380	75	25	100	2110
530	JSS 2	Low	17.3	1700	155	60	300	1235	50	270	320	2065
531	SS 2	Low	17.51	1530	500	50	300	1270	450	10	460	2230
532	SS 2	Low	17.18	1530	280	420	50	1270	80	160	240	2255
533	SS 2	Low	16.76	1540	305	420	80	1235	130	180	310	2305
534	JSS 2	Low	18.52	1550	90	150	0	1280	60	0	60	1790
535	JSS 3	Low	16.66	1575	210	450	300	1340	70	75	145	2385
536	JSS 3	Low	16.18	1570	345	210	600	1410	95	110	205	2425
537	SS 1	Low	17.48	1800	230	120	600	1270	70	390	460	2450
538	SS 3	Low	16.14	1740	200	600	0	1080	480	140	620	2540
539	SS 1	Low	17.85	1800	700	10	600	1270	420	510	930	2810
540	SS 1	Low	18.29	1600	630	480	200	1270	140	520	660	2810
541	SS 2	Low	22.48	1545	750	540	100	1500	220	275	495	2885
542	SS 2	Low	15.76	1800	527	320	150	1422	480	275	755	2797
543	JSS 2	Low	17.44	1565	550	120	300	1210	185	420	605	2385
544	SS 2	Low	17.67	1770	1680	300	0	1620	675	855	1530	3750
545	SS 2	Low	18.04	1570	1230	560	100	1320	920	260	1180	3410
546	SS 2	Low	16.96	1550	1210	900	100	1740	520	200	720	3710
547	SS 2	Low	16.76	1595	1500	840	0	2040	645	110	755	3780
548	JSS 2	Low	18.75	1425	1680	945	missing	1610	1070	915	1985	3780
549	SS 1	Low	19.14	1675	130	840	0	1235	110	160	270	2645
550	JSS 2	Low	26.04	1550	105	300	125	1235	120	0	120	2055
551	JSS 3	Low	20.08	1600	490	0	300	1305	425	60	485	2240
552	JSS 2	Low	18.94	0	0	0	0	0	0	0	0	0
553	SS 3	Low	20.45	1500	0	0	150	1200	0	0	0	1575
554	SS 1	Low	24.84	800	412	300	300	642	380	190	570	1662
555	SS 3	Low	22.86	1500	60	420	0	1200	0	60	60	1980
556	JSS 2	Low	24.44	1510	400	420	0	1410	80	120	200	2330
557	SS 1	Low	22.15	1800	125	420	200	1235	300	90	390	2445

558	JSS 3	Low	18.82	1655	175	630	0	1235	110	185	295	2460
559	JSS 3	Low	20.27	1650	1680	0	200	1830	940	470	1410	3430
560	JSS 2	Low	20.06	1800	1680	1260	100	2100	845	150	995	3780
561	SS 3	Low	22.04	1550	30	420	130	1200	50	30	80	2050
562	SS 3	Low	19.38	1660	220	180	300	1360	60	160	220	2210
563	JSS 2	Low	21.14	1550	1260	360	0	2040	50	420	470	3170
564	JSS 2	Low	20.96	150	1680	750	300	630	630	780	1410	2730
565	SS 1	Low	21.01	700	125	630	150	435	300	90	390	1530
566	SS 1	Low	22.53	1520	60	0	0	1200	20	60	80	1580
567	SS 3	Low	22.49	1500	120	0	0	1200	30	90	120	1620
568	SS 3	Low	17.51	1500	0	90	150	1200	0	0	0	1665
569	SS 3	Low	20.05	1550	295	0	0	1270	155	120	275	1845
570	SS 2	Low	19.03	1587	70	150	100	1220	12	125	137	1857
571	SS 1	Low	21.01	1500	0	300	300	1200	0	0	0	1950
572	SS 1	Low	22.05	1650	75	30	400	1200	35	190	225	1955
573	SS 2	Low	20.96	1520	560	80	0	1650	60	70	130	2160
574	SS 3	Low	19.15	1525	600	0	100	1800	25	0	25	2175
575	SS 2	Low	20.7	1500	700	0	0	1480	0	420	420	2200
576	SS 1	Low	20.44	1800	420	0	0	1200	360	360	720	2220
577	SS 3	Low	18.62	1500	805	0	0	1305	70	630	700	2305
578	JSS 2	Low	20.03	1550	335	360	170	1410	75	100	175	2445
579	JSS 2	Low	22.2	1700	409	360	0	1560	50	199	249	2469
580	SS 3	Low	25.78	1185	390	840	150	1110	195	270	465	2490
581	SS 3	Low	19	1800	770	0	0	1340	360	620	980	2570
582	JSS 2	Low	20.57	1590	560	450	300	1410	180	260	440	2750
583	SS 1	Low	20.28	1530	210	840	600	1200	30	210	240	2880
584	SS 2	Low	19.35	1540	689	630	80	1690	229	10	239	2899
585	SS 2	Low	19.04	1510	595	840	60	1305	430	70	500	2975
586	SS 2	Low	19.04	1520	475	840	300	1200	300	195	495	2985
587	SS 2	Low	19.87	1525	1130	400	0	1620	375	360	735	3055
586	JSS 3	Low	18.65	1575	1470	120	150	2040	420	285	705	3240
587	SS 1	Low	19.49	1650	1680	0	200	2040	840	150	990	3430
588	SS 3	Low	29.67	1550	60	0	150	1200	50	60	110	1685
589	SS 2	Low	27.55	1550	270	900	0	1410	0	110	110	2720
590	SS 2	Low	27.34	1500	1050	50	200	1620	450	180	630	2700
591	JSS 2	Low	30.86	1700	1680	180	600	1620	1100	360	1460	3780
592	SS 3	Low	33.02	1550	180	0	300	1200	50	180	230	1880
593	SS 1	Low	16.53	300	600	900	100	300	0	600	600	1800
594	JSS 3	Low	15.62	1570	365	400	300	1235	230	170	400	2485
595	JSS 2	Low	15.82	1800	1500	480	900	2100	750	750	1500	3780
596	JSS 2	Low	16.65	180	300	150	0	175	170	135	305	630
597	JSS 2	Low	15.81	190	500	150	0	375	170	145	315	840
598	JSS 2	Low	18.37	0	145	600	125	70	0	75	75	795

599	SS 1	Low	17.78	1500	10	0	0	1200	0	10	10	1510
600	SS 1	Low	16.89	1530	120	0	100	1200	60	90	150	1750
601	SS 2	Low	17.02	1610	175	75	0	1250	150	85	235	1860
602	SS 1	Low	17.93	1500	50	420	0	1200	10	40	50	1970
603	SS 1	Low	17.29	725	972	240	150	642	865	190	1055	2087
604	SS 1	Low	16.87	1650	275	360	0	1235	290	100	390	2285
605	JSS 3	Low	16.65	1531	455	240	0	1225	16	445	461	2226
606	JSS 2	Low	17.8	1510	535	360	0	1235	430	80	510	2405
607	JSS 3	Low	15.31	1555	290	720	0	1200	215	130	345	2565
608	JSS 2	Low	17.71	1560	110	300	0	1270	10	90	100	1970
609	JSS 3	Low	17.12	1610	420	450	400	1340	80	310	390	2680
610	JSS 2	Low	17.01	1060	360	540	900	990	100	330	430	2710
611	JSS 2	Low	17.85	1571	350	600	600	1400	120	101	221	2821
612	JSS 2	Low	18.29	1800	700	30	300	1270	315	915	1230	2680
613	JSS 3	Low	16.85	1500	660	1295	0	1830	0	30	30	3455
614	JSS 3	Low	16.82	1535	620	1260	0	1620	60	175	235	3415
615	JSS 3	Low	18.31	1607	1230	720	0	1620	637	280	917	3557
616	SS 1	Low	16.89	1625	1680	60	100	2100	560	825	1385	3465
617	JSS 2	Low	17.31	1580	1680	840	110	2100	85	870	955	3780
618	SS 3	Low	18.82	1500	100	0	0	1235	35	30	65	1600
619	SS 3	Low	18.59	1500	140	180	0	1235	15	90	105	1820
620	JSS 3	Low	16.88	1565	325	770	0	1300	120	170	290	2660
621	SS 2	Low	17.58	1610	650	420	0	1270	620	70	690	2680
622	JSS 2	Low	16.97	1655	710	840	100	1350	425	290	715	3255
623	SS 3	Low	16.04	0	511	360	150	49	42	420	462	946
624	JSS 2	Low	16.51	200	259	525	0	124	260	75	335	984
625	SS 1	Low	18.75	1510	21	0	0	1200	31	0	31	1531
626	SS 1	Low	18.13	1500	30	0	0	1200	0	30	30	1530
627	SS 3	Low	18	1500	100	0	120	1235	35	30	65	1660
628	SS 3	Low	17.91	1500	105	0	200	1200	35	70	105	1705
629	JSS 3	Low	16.14	1510	85	210	100	1270	10	15	25	1855
630	SS 3	Low	16.71	1545	280	180	0	1270	90	165	255	2005
631	JSS 3	Low	18.38	1535	161	315	0	1284	45	67	112	2011
632	JSS 3	Low	15.55	1662	140	240	0	1235	82	185	267	2042
633	JSS 3	Low	16.94	1710	235	30	75	1340	80	225	305	2050
634	JSS 3	Low	16.22	1615	245	120	200	1270	110	180	290	2080
635	JSS 3	Low	17.96	1700	105	300	0	1235	170	100	270	2105
636	SS 3	Low	18.37	1580	340	120	150	1260	160	200	360	2190
637	JSS 2	Low	16.18	1650	25	0	0	1225	0	150	150	1675
638	JSS 3	Low	14.42	1555	105	630	0	1235	40	85	125	2290
639	JSS 3	Low	18.78	1560	539	210	100	1410	129	260	389	2359
640	SS 2	Low	18.69	1520	0	840	0	1200	20	0	20	2360
641	JSS 3	Low	17.3	1545	150	600	200	1230	105	60	165	2395

642	JSS 2	Low	18.31	1510	80	60	300	1270	10	10	20	1800
643	JSS 3	Low	18.44	1670	154	315	600	1235	90	199	289	2439
644	SS 1	Low	18.37	1675	35	750	0	1235	75	100	175	2460
645	SS 1	Low	16.36	1700	300	450	100	1300	200	200	400	2550
646	JSS 2	Low	15.7	1800	240	0	200	1250	110	190	300	2140
647	JSS 2	Low	17.37	1535	210	120	200	1200	230	15	245	1965
648	SS 2	Low	16.23	1560	175	840	0	1235	80	120	200	2575
649	SS 2	Low	17.57	1562	475	635	60	1480	117	140	257	2702
650	JSS 3	Low	15.01	1555	375	840	0	1305	215	110	325	2770
651	JSS 2	Low	18.73	1555	560	75	60	1240	425	150	575	2220
652	SS 3	Low	18.26	1770	690	120	0	1290	270	600	870	2580
653	JSS 3	Low	16.8	1560	385	945	150	1410	115	120	235	2965
654	JSS 2	Low	17.36	1800	290	240	0	1225	185	450	635	2330
655	JSS 3	Low	16.41	1585	700	630	150	1690	35	260	295	3065
656	JSS 3	Low	16.82	1660	1320	540	0	1500	610	570	1180	3520
657	JSS 2	Low	17.26	1735	384	840	60	1224	190	405	595	2989
658	JSS 3	Low	17.51	1650	875	1080	0	2040	35	150	185	3605
659	JSS 2	Low	18.29	1800	840	420	150	1500	500	540	1040	3135
660	SS 2	Low	16.56	1560	1680	630	0	1830	850	350	1200	3780
661	JSS 3	Low	18.34	1735	1200	510	100	1380	975	280	1255	3545
662	JSS 3	Low	15.19	1525	1460	360	200	1620	490	575	1065	3445
663	JSS 2	Low	16.33	1540	1650	840	0	1290	1600	300	1900	3780
664	SS 2	Low	18.26	1540	1560	360	0	1620	860	320	1180	3460
665	SS 2	Low	17.87	1560	2050	630	0	1830	1130	350	1480	3780
666	SS 2	Low	18.19	1690	1680	0	600	2100	600	270	870	3780
667	JSS 2	Low	21.72	380	440	450	200	460	310	50	360	1370
668	SS 1	Low	23.5	600	720	0	0	1080	240	0	240	1320
669	JSS 3	Low	20.94	1605	100	450	0	1235	40	130	170	2155
670	JSS 2	Low	19.72	1530	250	180	100	1200	15	265	280	2010
671	JSS 3	Low	22.32	1710	183	200	60	1233	150	210	360	2123
672	SS 2	Low	25.06	1710	30	840	0	1230	160	50	210	2580
673	JSS 2	Low	19.34	1580	840	150	0	1620	215	285	500	2570
674	JSS 2	Low	19.47	1730	1680	840	100	1920	460	890	1350	3780
675	JSS 2	Low	19.03	380	130	180	600	350	15	145	160	990
676	JSS 2	Low	22.49	200	1680	540	300	420	1100	360	1460	2570
677	SS 1	Low	22.31	1525	135	90	0	1235	95	30	125	1750
678	JSS 3	Low	19.03	1650	275	0	0	1400	120	105	225	1925
679	SS 1	Low	21.94	1500	70	360	0	1270	0	0	0	1930
680	JSS 3	Low	21.78	1620	100	135	200	1235	55	130	185	1955
681	SS 1	Low	19.26	1575	294	120	150	1249	140	180	320	2064
682	SS 3	Low	21.08	1500	0	630	100	1200	0	0	0	2180
683	JSS 2	Low	20.32	1580	700	300	400	1235	85	660	745	2780
684	JSS 2	Low	20.54	1510	935	540	0	1740	45	360	405	2985

685	JSS 2	Low	20.58	1700	780	360	300	1410	200	570	770	2990
686	JSS 2	Low	24.65	1600	950	420	100	1250	650	350	1000	3020
687	SS 3	Low	20.58	1500	0	0	100	1200	0	0	0	1550
688	SS 3	Low	19.29	1500	0	0	150	1200	0	0	0	1575
689	SS 3	Low	20.29	1650	0	0	0	1200	0	150	150	1650
690	SS 3	Low	25.95	1500	0	360	300	1200	0	0	0	2010
691	SS 3	Low	23.31	1550	60	420	300	1200	50	60	110	2180
692	SS 3	Low	20.2	1500	335	360	0	1235	90	210	300	2195
693	SS 3	Low	22.49	1610	720	0	150	1560	460	10	470	2405
694	JSS 2	Low	21.19	1655	115	180	0	1210	20	240	260	1950
695	SS 3	Low	24.21	1260	960	630	0	1020	900	120	1020	2850
696	SS 1	Low	22.04	1630	840	720	0	1200	420	550	970	3190
697	JSS 2	Low	19.38	325	420	630	0	460	50	235	285	1375
698	JSS 3	Low	22.86	1500	0	0	0	1200	0	0	0	1500
699	SS 3	Low	20.69	1500	105	0	150	1225	20	60	80	1680
700	SS 3	Low	24.84	1500	370	50	0	1320	70	180	250	1920
701	SS 1	Low	21.16	1650	190	45	40	1225	255	60	315	1905
702	SS 2	Low	20.69	1605	75	300	0	1215	30	135	165	1980
703	SS 3	Low	25.25	1500	335	240	0	1235	0	300	300	2075
704	SS 1	Low	20.82	1500	585	0	0	1235	60	490	550	2085
705	SS 1	Low	20.28	1500	135	120	700	1200	35	100	135	2155
706	SS 2	Low	19.92	1610	560	0	0	1270	270	330	600	2170
707	JSS 3	Low	19.84	1554	217	350	100	1277	39	155	194	2171
708	SS 2	Low	25.1	1600	395	240	0	1410	50	235	285	2235
709	SS 2	Low	20.05	1650	210	360	300	1200	0	360	360	2370
710	SS 2	Low	21.97	1600	570	105	100	1410	0	460	460	2375
711	SS 2	Low	22.52	1680	630	0	0	1560	190	260	450	2310
712	JSS 2	Low	19.96	1500	15	384	0	1215	0	0	0	1899
713	SS 3	Low	22.21	1650	30	840	0	1200	0	180	180	2520
714	JSS 2	Low	19.61	1500	35	240	50	1235	0	0	0	1825
715	SS 2	Low	21.91	1550	240	540	200	1200	0	290	290	2430
716	SS 3	Low	19.82	1500	335	480	100	1290	35	210	245	2365
717	SS 3	Low	22.76	1800	380	150	150	1200	470	300	770	2480
718	SS 2	Low	20.08	1610	850	270	0	1270	430	460	890	2730
719	JSS 2	Low	20.58	1700	850	360	300	1480	200	570	770	3060
720	SS 1	Low	21.3	1530	1260	280	0	1200	1260	0	1260	3070
721	SS 2	Low	19.61	1510	840	840	0	1200	10	840	850	3190
722	SS 2	Low	19.37	1800	180	900	300	1200	190	330	520	3180
723	SS 2	Low	19.05	1610	860	750	70	1550	480	140	620	3255
724	SS 3	Low	19.47	1590	1410	360	0	2040	210	450	660	3360
725	JSS 2	Low	23.73	1800	840	420	160	1500	500	540	1040	3185
726	SS 2	Low	18.83	1610	1645	630	100	1830	585	540	1125	3780
727	JSS 2	Low	22.49	1645	610	896	600	1620	190	145	335	3451

728	JSS 2	Low	20.42	1610	1660	420	0	2100	370	140	510	3690
729	SS 3	Low	28.76	1500	360	600	0	1560	0	0	0	2460
730	SS 3	Low	30.06	1500	0	240	0	1200	0	0	0	1740
731	SS 3	Low	20.75	1500	230	320	0	1270	70	90	160	2050
732	SS 1	Low	28.72	1650	720	900	300	1800	60	210	270	3420
733	JSS 2	Low	34.48	1600	1035	450	100	1290	155	890	1045	3135
734	JSS 2	Medium	17.3	0	50	420	100	0	75	25	100	520
735	JSS 2	Medium	16.85	1510	160	180	0	1200	80	90	170	1850
736	JSS 3	Medium	17.67	1555	1440	600	100	1620	425	650	1075	3645
737	SS 3	Medium	18.75	1800	90	240	200	1200	300	90	390	2230
738	JSS 2	Medium	15.61	1575	305	720	0	1410	110	60	170	2600
739	SS 1	Medium	18.91	1675	575	240	300	1235	445	270	715	2640
740	JSS 3	Medium	14.38	1550	1050	210	70	1620	210	470	680	2845
741	SS 2	Medium	16.33	1590	428	660	100	1480	90	148	238	2728
742	JSS 2	Medium	17.36	1510	260	840	300	1270	20	180	200	2910
743	JSS 3	Medium	15.4	1650	1500	0	0	2040	420	390	810	3150
744	JSS 3	Medium	17.4	1675	1410	450	300	2040	235	510	745	3685
745	JSS 2	Medium	17.85	1510	625	1050	150	1335	360	140	500	3335
746	SS 2	Medium	18.44	1660	300	300	150	1350	20	290	310	2410
747	SS 2	Medium	17.51	1530	580	180	175	1480	240	90	330	2465
748	SS 2	Medium	18.52	1580	407	450	100	1235	210	242	452	2487
749	SS 2	Medium	18.38	1590	380	680	50	1380	100	190	290	2700
750	JSS 3	Medium	17.58	1650	675	1050	0	1515	210	300	510	3375
751	SS 2	Medium	18.59	1550	570	1080	0	1410	0	410	410	3200
752	SS 3	Medium	14.86	1500	900	0	300	1200	630	270	900	2550
753	SS 2	Medium	17.84	1520	1380	360	100	1410	1060	130	1190	3310
754	SS 2	Medium	15.64	1580	1150	840	150	1690	510	230	740	3720
755	SS 2	Medium	16.85	1670	1680	840	300	1830	230	1200	1430	3780
756	JSS 2	Medium	21.88	1680	460	240	200	1440	330	70	400	2480
757	SS 2	Medium	19.82	1280	160	0	20	1230	100	60	160	1450
758	JSS 3	Medium	18.92	1500	0	0	0	1200	0	0	0	1500
759	JSS 3	Medium	22.86	1500	0	0	200	1200	0	0	0	1700
760	SS 2	Medium	24.22	1560	400	0	0	1270	240	150	390	1960
761	JSS 3	Medium	19.29	1560	190	900	100	1260	80	110	190	2700
762	JSS 2	Medium	19.05	1470	600	840	0	1290	270	510	780	2910
763	JSS 2	Medium	24.24	1830	1000	180	0	1360	450	720	1170	3010
764	JSS 3	Medium	19.05	1800	210	600	600	1410	480	180	660	2910
765	JSS 3	Medium	23.51	1650	1140	900	0	1410	840	240	1080	3690
766	SS 2	Medium	21.16	1800	1260	840	0	1620	720	420	1140	3780
767	SS 3	Medium	21.26	600	385	840	300	480	25	480	505	1975
768	SS 3	Medium	20.75	1575	30	120	120	1200	75	30	105	1785
769	SS 3	Medium	20.52	1900	0	0	0	1200	100	300	400	1900
770	SS 3	Medium	22.16	1200	140	600	0	960	140	0	140	1940

771	SS 3	Medium	20.15	1500	615	120	0	1305	210	300	510	2235
772	SS 2	Medium	19.82	1280	160	840	10	1230	100	60	160	2285
773	SS 2	Medium	20.15	1615	150	450	150	1200	40	225	265	2290
774	JSS 2	Medium	19.15	1550	270	150	80	1350	170	0	170	2010
775	SS 3	Medium	20.76	1240	1320	30	150	1410	505	645	1150	2665
776	SS 3	Medium	19.92	1800	630	210	0	1270	290	620	910	2640
777	SS 2	Medium	26.29	1605	480	450	500	1260	270	255	525	2785
778	JSS 2	Medium	19.37	1800	1680	840	0	1890	1615	400	2015	3780
779	JSS 2	Medium	18	390	0	120	0	350	20	20	40	510
780	JSS 3	Medium	17.78	90	292	840	0	120	225	37	262	1222
781	JSS 2	Medium	16.42	1800	0	180	220	1200	660	450	1110	2080
782	SS 1	Medium	17.78	1580	0	360	0	1200	80	0	80	1940
783	SS 1	Medium	16.44	900	630	270	0	660	270	600	870	1800
784	JSS 2	Medium	17.63	1530	155	540	0	1205	15	165	180	2225
785	JSS 2	Medium	16.41	1655	220	210	300	1235	40	300	340	2235
786	JSS 2	Medium	17.93	1510	1155	180	0	1235	500	630	1130	2845
787	JSS 3	Medium	17.65	1585	1330	130	0	1830	605	180	785	3045
788	JSS 3	Medium	18.02	1560	440	840	300	1380	150	170	320	2990
789	JSS 3	Medium	18.56	1670	1435	420	300	1760	55	990	1045	3675
790	JSS 2	Medium	18.9	1160	1327	180	900	1027	1030	430	1460	3242
791	SS 2	Medium	18.37	1800	1500	600	100	1800	1000	550	1550	3780
792	SS 1	Medium	16.46	485	420	0	150	435	45	425	470	980
793	SS 1	Medium	17.56	1620	0	0	300	1200	120	0	120	1770
794	JSS 3	Medium	18.73	1660	90	40	0	1245	45	160	205	1790
795	JSS 3	Medium	17.72	1525	133	180	0	1235	35	88	123	1838
796	JSS 3	Medium	15.24	1560	195	90	0	1225	150	80	230	1845
797	JSS 2	Medium	12.74	1510	100	240	0	1230	40	40	80	1850
798	JSS 2	Medium	17.98	1550	260	0	100	1410	75	25	100	1860
799	JSS 3	Medium	16.87	1700	85	90	300	1210	85	190	275	2025
800	JSS 3	Medium	14.22	1553	295	180	0	1340	53	155	208	2028
801	JSS 3	Medium	13.5	1540	155	420	100	1290	50	55	105	2165
802	JSS 2	Medium	15.31	1505	405	75	200	1410	180	20	200	2185
803	JSS 3	Medium	16.81	1540	130	600	0	1200	85	85	170	2270
804	JSS 3	Medium	18.44	1535	105	630	100	1235	30	75	105	2320
805	JSS 3	Medium	17.51	1605	305	420	0	1235	90	285	375	2330
806	SS 1	Medium	17.72	1750	360	180	85	1560	150	100	250	2375
807	JSS 2	Medium	16.94	1700	540	0	300	1560	260	120	380	2390
808	JSS 3	Medium	17.44	1570	240	600	0	1200	20	290	310	2410
809	JSS 2	Medium	14.84	1660	90	420	100	1200	40	210	250	2220
810	JSS 3	Medium	13.62	1610	405	630	0	1225	150	340	490	2645
811	JSS 3	Medium	15.01	1754	684	60	500	1249	39	850	889	2748
812	JSS 3	Medium	16.61	1605	960	120	480	1500	265	500	765	2925
813	JSS 2	Medium	16.56	1600	650	600	80	1500	100	300	400	2950

814	JSS 2	Medium	16.87	1575	25	360	900	1200	50	50	100	2810
815	JSS 3	Medium	14.57	1605	1435	90	200	1935	600	205	805	3230
816	SS 2	Medium	18.67	1800	1050	360	0	1200	1030	420	1450	3210
817	JSS 3	Medium	16.02	1535	1560	0	0	1620	850	325	1175	3095
818	JSS 3	Medium	13.33	1650	1500	900	0	2100	240	150	390	3780
819	JSS 2	Medium	17.36	1650	1680	0	0	2100	420	990	1410	3330
820	JSS 3	Medium	16.02	1550	30	180	100	1200	50	30	80	1810
821	JSS 3	Medium	18.14	1500	1050	0	0	1620	420	210	630	2550
822	SS 3	Medium	18.14	1500	245	840	0	1270	35	140	175	2585
823	SS 2	Medium	16.18	1580	335	630	100	1290	230	95	325	2595
824	JSS 2	Medium	15.82	1510	380	840	0	1230	150	210	360	2730
825	SS 2	Medium	15.21	1515	864	630	150	1800	39	240	279	3109
826	SS 3	Medium	18.59	1500	0	270	0	1200	0	0	0	1770
827	SS 3	Medium	17.65	1500	67	180	150	1200	35	32	67	1822
828	SS 3	Medium	18.42	1500	180	630	0	1200	0	180	180	2310
829	JSS 2	Medium	16.9	1550	245	120	150	1245	130	120	250	1990
830	SS 2	Medium	18.59	1680	330	480	240	1340	150	220	370	2610
831	SS 2	Medium	14.69	1580	515	450	100	1235	150	410	560	2595
832	JSS 3	Medium	16.44	1579	202	1260	300	1252	154	75	229	3191
833	JSS 3	Medium	17.42	1600	1380	360	0	2040	470	170	640	3340
834	JSS 3	Medium	16.22	1555	1565	630	0	1740	460	620	1080	3750
835	SS 2	Medium	17.78	1600	1295	840	300	1235	420	940	1360	3780
836	JSS 3	Medium	16.4	1650	1680	900	0	2100	840	240	1080	3780
837	JSS 2	Medium	17.63	1506	690	80	missing	1260	276	360	636	2276
838	JSS 2	Medium	16.42	1510	181	360	0	1226	45	120	165	2051
839	JSS 2	Medium	19.49	1575	1530	840	0	1830	350	625	975	3780
840	SS 3	Medium	19.29	1600	114	120	100	1224	150	40	190	1884
841	SS 2	Medium	22.05	1590	480	0	0	1410	150	210	360	2070
842	JSS 3	Medium	21.36	1535	290	360	350	1270	220	35	255	2360
843	JSS 3	Medium	19.23	1800	215	90	0	1200	60	780	840	2105
844	SS 1	Medium	20.17	1250	840	600	100	1200	720	120	840	2740
845	JSS 3	Medium	21.98	1700	600	600	200	1200	780	20	800	3000
846	SS 2	Medium	21.71	1800	450	150	150	1200	330	510	840	2550
847	SS 3	Medium	19.96	1800	1730	240	200	2100	1040	330	1370	3780
848	JSS 3	Medium	23.44	1500	1680	630	600	2100	35	1260	1295	3780
849	SS 2	Medium	22.99	1600	105	450	0	1235	35	135	170	2155
850	SS 2	Medium	20.99	1570	480	60	0	1410	100	240	340	2110
851	SS 3	Medium	22.5	1500	0	630	0	1200	0	0	0	2130
852	SS 2	Medium	22.76	1730	390	180	0	1320	200	300	500	2300
853	SS 2	Medium	20.38	1800	450	180	120	1410	150	480	630	2490
854	SS 2	Medium	25.85	1700	120	840	0	1200	170	150	320	2660
855	SS 3	Medium	21.63	1500	745	420	150	1225	90	630	720	2740
856	SS 3	Medium	19.82	1500	0	0	150	1200	0	0	0	1575

857	SS 2	Medium	24.24	1530	60	30	150	1200	30	60	90	1695
858	JSS 2	Medium	19.98	325	65	840	0	285	50	55	105	1230
859	SS 3	Medium	19.82	1500	70	180	0	1270	0	0	0	1750
860	SS 2	Medium	19.84	1550	175	30	0	1340	0	85	85	1755
861	SS 3	Medium	23.15	1650	90	180	0	1290	150	0	150	1920
862	SS 3	Medium	25.54	1625	0	20	600	1200	50	75	125	1945
863	SS 1	Medium	19.94	1500	0	360	200	1200	0	0	0	1960
864	SS 3	Medium	19.96	1800	45	180	0	1245	90	240	330	2025
865	SS 2	Medium	24.41	1530	0	630	0	1200	30	0	30	2160
866	SS 2	Medium	25.1	1600	330	240	0	1410	100	120	220	2170
867	SS 2	Medium	21.63	1500	120	600	0	1200	0	120	120	2220
868	SS 3	Medium	20.11	1500	615	420	0	1305	210	300	510	2535
869	SS 2	Medium	19.83	1592	720	420	100	1250	462	300	762	2782
870	SS 1	Medium	20.08	1625	920	120	250	1340	420	485	905	2790
871	SS 2	Medium	22.58	1610	970	100	0	1270	430	580	1010	2680
872	SS 3	Medium	19.03	1650	1080	600	400	1800	460	170	630	3530
873	SS 2	Medium	20.6	1680	1260	180	0	1500	920	220	1140	3120
874	SS 1	Medium	21.2	1525	1075	630	300	1225	210	865	1075	3380
875	JSS 3	Medium	21.77	1800	1470	6	0	1620	930	430	1360	3276
876	SS 2	Medium	19.41	1800	1680	1050	150	1650	915	870	1785	3780
877	JSS 2	Medium	19.61	1800	1680	1050	300	2100	890	510	1400	3780
878	SS 2	Medium	26.87	1525	270	240	100	1340	0	155	155	2085
879	JSS 2	Medium	33.3	1800	420	840	0	1620	0	300	300	3060
880	SS 3	Medium	32.11	1500	330	0	0	1200	210	120	330	1830
881	JSS 2	High	13.85	1020	560	450	0	1110	260	210	470	2030
882	SS 2	High	18.25	190	370	910	0	290	100	170	270	1470
883	JSS 2	High	14.1	1550	0	0	0	1200	0	50	50	1550
884	JSS 3	High	14.57	1650	1680	360	200	2100	680	280	960	3780
885	JSS 2	High	16.44	1550	740	770	900	1200	290	500	790	3780
886	SS 2	High	17.37	1500	0	0	0	1200	0	0	0	1500
887	SS 2	High	17.97	1800	1080	240	900	1260	495	900	1395	3720
888	JSS 2	High	18.07	1755	1680	840	500	2040	845	500	1345	3780
889	JSS 3	High	25.82	1660	460	60	0	1410	220	190	410	2180
890	SS 2	High	22.41	1590	720	180	0	1200	720	90	810	2490
891	SS 3	High	19.26	1580	70	840	0	1235	115	0	115	2490
892	SS 3	High	21.77	1550	295	840	0	1305	70	170	240	2685
893	SS 3	High	21.77	1550	1225	840	0	1305	70	1100	1170	3615
894	SS 2	High	21.51	1500	315	0	200	1340	70	105	175	1915
895	SS 2	High	19.84	1550	140	360	0	1340	50	0	50	2050
896	SS 2	High	19.54	1540	525	270	0	1410	115	240	355	2335
897	JSS 2	High	15.22	1600	85	240	100	1210	125	50	175	1975
898	JSS 2	High	18.03	1600	120	120	100	1270	100	50	150	1890
899	JSS 2	High	15.01	1510	200	240	0	1220	70	120	190	1950

900	JSS 3	High	17.22	1550	380	180	0	1265	95	270	365	2110
901	JSS 3	High	15.27	1585	510	180	0	1655	70	70	140	2275
902	SS 3	High	13.02	1525	480	720	0	1200	420	85	505	2725
903	JSS 2	High	15.52	1650	420	630	100	1270	210	290	500	2750
904	JSS 3	High	17.93	1725	925	0	0	1225	990	135	1125	2650
905	JSS 2	High	18.36	1650	360	900	0	1380	330	0	330	2910
906	JSS 3	High	17.29	1590	980	735	0	1305	330	635	965	3305
907	JSS 3	High	16.94	1710	600	600	300	1380	430	200	630	3060
908	JSS 3	High	17.16	1530	1290	480	0	1740	395	385	780	3300
909	JSS 2	High	18.21	1800	890	180	170	2040	40	615	655	2995
910	JSS 2	High	16.17	1800	580	600	100	1410	350	620	970	3030
911	JSS 2	High	17.8	915	45	210	600	910	15	35	50	1470
912	SS 2	High	17.36	1560	75	0	100	1200	15	120	135	1695
913	SS 2	High	16.6	1500	0	300	0	1200	0	0	0	1800
914	JSS 2	High	13.78	1520	490	70	0	1410	90	210	300	2080
915	JSS 2	High	17.01	1510	220	300	0	1230	80	120	200	2030
916	JSS 3	High	17.63	1675	50	420	0	1235	75	115	190	2145
917	JSS 3	High	17.85	1582	530	180	0	1655	67	90	157	2292
918	JSS 3	High	18.9	1555	520	300	0	1380	245	150	395	2375
919	JSS 2	High	17.15	1740	480	180	0	1500	330	90	420	2400
920	JSS 2	High	15.62	1655	112	240	300	1235	40	192	232	2157
921	JSS 2	High	15.27	1510	680	270	0	1725	45	120	165	2460
922	JSS 2	High	16.02	1530	455	630	0	1410	65	210	275	2615
923	SS 2	High	18.93	1800	990	0	0	1900	540	150	690	2790
924	JSS 2	High	15.15	1540	1200	150	0	1620	445	375	820	2890
925	JSS 2	High	14.67	1510	1470	180	0	1620	430	630	1060	3160
926	JSS 2	High	15.01	1655	965	90	300	2040	40	240	280	2860
927	JSS 2	High	15.56	1625	840	420	150	1500	500	165	665	2960
928	JSS 2	High	17.48	1600	1320	600	0	1260	1050	310	1360	3520
929	JSS 2	High	18.83	1550	360	840	300	1200	140	270	410	2900
930	JSS 2	High	13.79	1510	1680	900	0	2100	190	240	430	3780
931	JSS 2	High	16.66	1800	1680	240	900	2100	930	720	1650	3780
932	SS 3	High	16.41	1500	130	360	0	1200	70	60	130	1990
933	SS 2	High	16.02	1500	40	840	0	1200	0	40	40	2380
934	SS 2	High	17.24	1535	365	240	0	1320	55	225	280	2140
935	JSS 2	High	16.53	1600	125	540	0	1305	50	70	120	2265
936	SS 2	High	16.33	1800	270	100	0	1470	0	480	480	2170
937	SS 2	High	15.23	1755	285	375	300	1275	285	180	465	2565
938	JSS 2	High	16.51	1700	550	840	0	1350	200	400	600	3090
939	SS 2	High	13.42	1800	300	1050	0	1270	290	240	530	3150
940	SS 3	High	20.6	1500	635	720	150	1200	35	600	635	2930
941	SS 2	High	17.63	1570	1680	630	0	1830	580	610	1190	3880
942	SS 2	High	15.24	1770	1550	600	125	1760	675	585	1260	3780

943	SS 1	High	17.51	1640	1080	840	100	1830	350	240	590	3660
944	JSS 2	High	15.82	1655	510	840	150	1350	285	230	515	3080
945	SS 1	High	16.51	1500	0	0	0	1200	0	0	0	1500
946	SS 2	High	17.1	1580	270	0	80	1410	120	20	140	1890
947	SS 2	High	16.37	1500	150	180	300	1260	0	90	90	1980
948	SS 2	High	17.16	1500	160	270	300	1250	50	60	110	2080
949	SS 3	High	18.59	1500	60	630	0	1200	0	60	60	2190
950	JSS 2	High	17.84	1610	20	120	60	1220	10	100	110	1780
951	SS 2	High	17.06	1520	350	480	100	1270	140	160	300	2400
952	SS 2	High	17.58	1720	810	210	0	1480	170	580	750	2740
953	JSS 3	High	18.29	1650	1050	120	0	2040	210	150	360	2820
954	SS 2	High	17.24	1560	1120	150	0	1760	500	120	620	2830
955	SS 2	High	17.21	1580	155	1050	100	1270	80	85	165	2835
956	SS 2	High	18.29	1580	1080	490	300	1410	270	680	950	3300
957	SS 2	High	16.44	1620	810	540	0	1200	720	210	930	2970
958	JSS 3	High	18.56	1500	1260	600	0	2040	420	0	420	3360
959	SS 2	High	18.73	1800	990	315	300	1950	435	630	1065	3255
960	SS 3	High	17.78	1500	940	300	900	1360	700	80	780	3540
961	SS 3	High	17.63	1500	1680	720	0	2100	480	60	540	3780
962	SS 2	High	16.6	1560	1860	910	0	1900	570	650	1220	3780
963	SS 2	High	19.23	1550	245	360	0	1305	190	0	190	2155
964	SS 2	High	19.23	1550	245	540	0	1305	190	0	190	2335
965	SS 2	High	19.81	1500	510	240	0	1500	210	0	210	2250
966	SS 1	High	20.76	920	110	840	0	800	30	20	50	1870
967	SS 2	High	21.77	1580	190	540	0	1270	120	80	200	2310
968	JSS 2	High	24.52	1650	160	360	60	1235	5	270	275	2200
969	SS 2	High	21.48	1580	140	840	200	1270	150	0	150	2660
970	SS 2	High	20.31	1740	840	120	0	1560	120	600	720	2700
971	JSS 3	High	19.05	1595	1070	210	0	1760	440	165	605	2875
972	JSS 2	High	19.38	1650	360	900	200	1380	330	0	330	3010
973	JSS 2	High	19.4	1575	985	360	100	1240	155	865	1020	2970
974	SS 2	High	19.53	1670	670	840	120	1690	250	100	350	3240
975	JSS 2	High	19.26	1535	565	540	450	1230	185	385	570	2865
976	SS 2	High	19.38	1500	1200	840	80	2040	0	360	360	3580
977	SS 2	High	21.85	1740	1680	720	300	2040	510	570	1080	3780
978	JSS 3	High	19.96	1585	130	80	100	1305	15	95	110	1820
979	JSS 3	High	22.86	1635	145	125	60	1225	60	195	255	1935
980	JSS 3	High	20.58	1655	150	180	0	1200	5	300	305	1985
981	JSS 2	High	20.07	1585	42	665	0	1200	10	117	127	2292
982	JSS 3	High	20.07	1575	930	121	200	1830	235	140	375	2726
983	SS 3	High	23.15	735	200	480	0	555	195	65	260	1415
984	SS 2	High	19.03	1550	290	0	100	1200	140	200	340	1890
985	SS 1	High	19.94	1500	220	60	360	1270	0	150	150	1960

986	SS 2	High	22.04	1580	290	0	200	1320	80	170	250	1970
987	SS 3	High	20	1560	485	630	0	1560	75	410	485	2675
988	SS 2	High	19.36	1570	980	450	100	1480	440	330	770	3050
989	SS 1	High	20.09	1740	720	480	250	1380	390	390	780	3190
990	JSS 2	High	19.11	1655	1020	420	0	1620	425	330	755	3095
991	SS 2	High	20.11	1580	1470	0	0	1410	920	420	1340	3050
992	SS 2	High	21.72	1630	1680	600	0	1935	920	470	1390	3780
993	JSS 2	High	19.29	1550	0	280	0	1200	0	50	50	1830
994	SS 2	High	19.38	1680	340	90	0	1200	430	90	520	2110
995	JSS 2	High	19.43	1500	335	240	0	1410	25	100	125	2075
996	SS 2	High	20.31	1600	270	360	100	1270	0	300	300	2280
997	SS 2	High	19.47	1575	282	900	0	1242	15	300	315	2757
998	SS 2	High	23.11	1600	490	840	0	1340	140	310	450	2930
999	JSS 3	High	19.07	1655	720	840	0	1620	215	240	455	3215
1000	JSS 3	High	20.55	1800	620	600	0	1410	590	335	925	3020
1001	SS 2	High	17.85	1590	795	1080	80	1250	740	95	835	3505
1002	SS 2	High	22.23	1590	984	210	100	1284	490	500	990	2834
1003	SS 2	High	23.12	1800	700	1050	400	1620	430	200	630	3750
1004	SS 2	High	28.51	1630	175	240	0	1200	105	200	305	2045
1005	SS 2	High	27.15	1800	1680	630	900	1830	710	1080	1790	3780
1006	SS 2	High	31.83	1605	660	720	0	1260	280	425	705	2985
